# Radio-metal cross-linking of alginate hydrogels for non-invasive *in vivo* imaging

**DOI:** 10.1016/j.biomaterials.2020.119930

**Published:** 2020-06

**Authors:** P. Stephen Patrick, Joseph C. Bear, Heather E. Fitzke, May Zaw-Thin, Ivan P. Parkin, Mark F. Lythgoe, Tammy L. Kalber, Daniel J. Stuckey

**Affiliations:** a Centre for Advanced Biomedical Imaging, Division of Medicine, University College London, London, WC1E 6DD, UK; b School of Life Science, Pharmacy & Chemistry, Kingston University, Penrhyn Road, Kingston upon Thames, KT1 2EE, UK; c Materials Chemistry Centre, Department of Chemistry, University College London, 20 Gordon Street, London, WC1H 0AJ, UK

**Keywords:** Biomaterials, Drug delivery, Stem cells, Tissue engineering, Hydrogels, Biological sciences, Medical sciences, SPECT

## Abstract

Alginate hydrogels are cross-linked polymers with high water content, tuneable chemical and material properties, and a range of biomedical applications including drug delivery, tissue engineering, and cell therapy. However, their similarity to soft tissue often renders them undetectable within the body using conventional bio-medical imaging techniques. This leaves much unknown about their behaviour *in vivo*, posing a challenge to therapy development and validation.

To address this, we report a novel, fast, and simple method of incorporating the nuclear imaging radio-metal ^111^In into the structure of alginate hydrogels by utilising its previously-undescribed capacity as an ionic cross-linking agent. This enabled non-invasive *in vivo* nuclear imaging of hydrogel delivery and retention across the whole body, over time, and across a range of model therapies including: nasal and oral drug delivery, stem cell transplantation, and cardiac tissue engineering. This information will facilitate the development of novel therapeutic hydrogel formulations, encompassing alginate, across disease categories.

## 1 Introduction

Alginate hydrogels are bio-compatible cross-linked polymers with high water content. They are tuneable in their mechanical and chemical properties [[1], [2], [3]], allowing versatile fabrication across the nano to macro scales [4] - from aerosols [5], micro-particles [6], micro-needles [7], capsules [8], and sheets [9], to 3D-printed shapes [10]. The biocompatibility and structure of alginate make it an effective support scaffold for bioelectronics [11], therapeutic cells and tissue engineering [12], and a versatile delivery vehicle for pro-biotics [13], small molecule drugs [14], genes [15], or proteins such as growth factors [16]. This flexibility has enabled alginate to become widespread in established consumer and medical products (Table S1), with new biomedical applications such as on-demand controllable drug release and origami-based tissue engineering [17] still emerging and yet to reach the clinic [8].

However, the similarity of standard Ca^2+^-cross-linked alginate hydrogels to soft tissue hinders their detection by the main medical imaging modalities Magnetic Resonance Imaging (MRI), X-ray Computed Tomography (CT), and ultrasound – thereby limiting understanding of the behaviour of implanted materials in animal models of disease and in patient populations. This lack of *in vivo* knowledge compromises development of new alginate-based therapies at both the preclinical phase, and during their translation in ongoing clinical trials (Table S2).

To address this problem, we took inspiration from the natural metal-chelating properties of alginate and developed a versatile method of cross-linking alginate hydrogels with radio-metal cations to enable non-invasive *in vivo* imaging with SPECT. Direct cross-linking of alginate with nuclear imaging isotopes has not been reported previously and offers a rapid and simple method for labelling hydrogels without altering their structure and properties. Using this method we show for the first time whole-body *in vivo* nuclear imaging of longitudinal retention and clearance of alginate hydrogels for oral and nasal drug delivery, stem cell transplantation, and cardiac tissue engineering.

We hope this labelling and imaging technique will inform upon dosing strategies, material optimisation, safety and efficacy of novel alginate-based therapies in biologically-relevant preclinical and clinical environments - hence accelerating their translation into well-characterised and safe therapeutic products. This method uses clinically-available materials and imaging techniques and so is readily translatable.

## 2 Alginate can be cross-linked by In^3+^ and Zr^4+^

Alginate is a seaweed-derived water-soluble linear co-polymer of mannuronic and guluronic acid. It can be cross-linked into a hydrogel using a solution of CaCl_2_ or BaCl_2_ which gelate via ionic bonding (Ca^2+^ or Ba^2+^) to negatively-charged carboxylates (COO^−^) across alginate polymers [1]. We tested the ability of InCl_3_ and ZrCl_4_ to cross-link alginate, as a route to imaging ^111^In and ^89^Zr with medical imaging modalities SPECT (Single-Photon-Emission-Computed-Tomography) and PET (Positron-Emission-Tomography) respectively. Unlike traditional metal-based radiolabelling which requires addition of an organic chelating group [18], direct incorporation of the radio-metal into the hydrogel structure simplifies labelling while demonstrating the hydrogel's structural integrity. The respective half-lives for ^111^In and ^89^Zr of 2.8 and 3.3 days permit repeat imaging over days to weeks - a time frame suitable for many biomedical applications.

To demonstrate In^3+^ and Zr^4+^ cross-linking, a 3% w/v sodium alginate solution was drop-cast into aqueous 300 mM InCl_3_ and ZrCl_4_ solutions. This produced hydrogel beads comparable in size and shape to those cross-linked with CaCl_2_ and BaCl_2_. X-ray Photoelectron Spectroscopy (XPS) analysis confirmed retention of the metals in the isolated hydrogel beads in only Ca^2+^, Ba^2+^ Zr^4+^ and In^3+^ oxidation states (Fig. S1 and Table S3). Analysis of alginate carbon binding energies using XPS confirmed In^3+^ and Zr^4+^ co-ordination with carboxyl and hydroxyl groups on the alginate sugar subunits (Fig. S2), as occurs with co-ordination of calcium [19]. Chelation was also confirmed by ATR-FTIR spectroscopy, as carboxyl, hydroxyl and ether linkages shifted considerably from the native sodium alginate (Fig. S3, Tables S4 and S5).

The ability of alginate to bind to and retain ^111^InCl_3_ and ^89^ZrCl_4_ was then measured with dialysis against a range of solutions. Retention of ^111^In in alginate dialysed against saline was good above and below physiological concentrations (0.9% w/v), with 75.8% ± 3.3 SD retention in 3% w/v alginate after 1 day (Figs. S4A and B), with an mean loss of 1.3% per day thereafter up to 8 days. Comparable retention of ^111^In in CaCl_2_ cross-linked hydrogel beads was also measured, with ~78% retained after 24 h of dialysis against physiological saline (Fig. S4C). In contrast ^111^InCl_3_ was rapidly lost from control saline solutions dialysed against saline, with only 14.7% ± 3.5 SD remaining after 24 h (Fig. S4C), demonstrating the role of alginate in retaining the metal.

Retention of ^111^InCl_3_ in 3% w/v alginate solutions dialysed against foetal bovine serum (FBS) showed a lower retention rate of 51.4% ± 1.7 SD at 24 h, with a mean loss of 3.8% per day thereafter up to 8 days (Fig. S4D). This suggests that cross-linking is less stable in the presence of biological molecules, but still sufficient to enable imaging over biologically-relevant timeframes. For example, this compares favourably to clinically-established ^111^In-oxine labelling of cells for SPECT imaging, which can lose as much as 70% of the label within the first 24 h [20]. To compare the strength of alginate binding to ^111^In with an established trivalent metal ion chelator, ^111^In-alginate was dialysed against 2.5 mM DTPA, giving retention in the dialysis membrane of 10.8% ± 1.7 SD after 24 h. This shows that though alginate chelates ^111^In^3+^, its binding is weaker than a purpose-designed chelator, as might be expected. Dialysis of ^111^In in 1% alginate against human serum gave retention of 62.1% ± 5.9 SD after 24 h, and 40.3% ± 4.3 SD after 7 days (Fig. S4E).

In comparison to ^111^In retention in alginate, ^89^Zr retention was highly variable and not reproducible. This suggests that though it is able to cross-link alginate in its Zr^4+^ form as shown by XPS (Figs. S1–3), cross-linking stability is less robust than that of ^111^In, and may be affected by zirconium's known pH and concentration-dependent hydrolytic conversion to zirconyl oxychloride [21], which may hinder its use for imaging alginate with PET in biological systems.

To demonstrate labelling stability under more physiological conditions, In^3+^ cross-linked alginate beads were implanted subcutaneously in mice and removed for analysis 4 and 10 days later (Fig. S5). At 4 days XPS showed the same characteristic carbon spectrum of the pre-implantation In-alginate beads in the *ex vivo* samples, with indium only in its In^3+^ state, showing maintained cross-linking. In samples excised after 10 days, XPS showed continued retention of indium in the sample, albeit as In_2_O_3_, with the carbon spectra suggesting calcium cross-linking rather than indium-cross-linking. Thus at later time points physiological calcium appears to replace indium as a cross-linker, with indium staying in the material as an insoluble oxide.

**Fig. 1:**
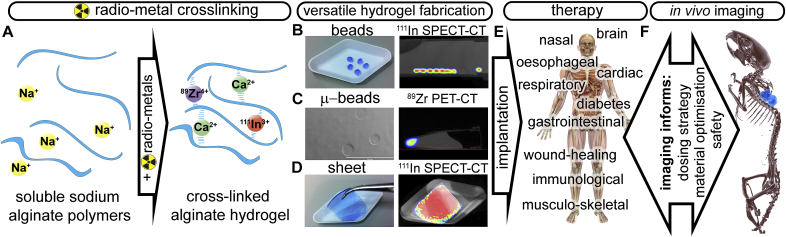
**A.** Schematic of alginate hydrogel cross-linking by calcium and radionuclides ^111^In^3+^ and ^89^Zr^4+^. Radiolabelled alginate can be made into **B.** beads, **C.** microbeads (scale bar 200 μm), and **D.** sheets, and imaged with SPECT-CT or PET-CT. **E.** Example therapy areas of hydrogel use **F.** 3D SPECT-CT rendering showing detection of ^111^In-alginate beads *in vivo*.

The versatility of the radiolabelling process was then demonstrated across a number of therapeutically-relevant formats. Alginate beads were synthesised using a drop-casting method, microbeads using spray fabrication, and sheets of alginate produced using a filter paper template (Fig. 1B–D). Macroscopic beads retained ^111^In activity over a week when implanted *in vivo* (Fig. S8).

Only sub-nanomole amounts of radio-metal ions need to be added to alginate solutions for *in vivo* detection, owing to their high specific activity (^111^In = 1720 MBq/nmol) and sensitivity of detection. Therefore, bulk physical properties of the hydrogel, including microstructure as observed using SEM (Fig. S6) and viscosity (Fig. S7), are not affected even when doped at an equivalent of 1 GBq/mL alginate. Even at this high concentration, around 10-fold higher than a realistic clinical dose, only 1 in 43194 alginate cross-linking sites would be occupied by ^111^In, assuming each metal ion binds two alginate sugar subunits.

We then demonstrated that our alginate radiolabelling method could be used to map the hydrogel in various clinically-relevant scenarios, including cardiac tissue engineering, stem cell delivery, and oral and nasal drug delivery.

## 3 Cardiac tissue engineering and stem cell implantation

Direct cardiac alginate injections are being evaluated as a mechanical support for modulation of ventricular remodelling after myocardial infarction in recent clinical trials [22,23]. Material implantation into the myocardial wall can be challenging due to pulsatile motion, therefore imaging has a clear role in informing on delivery success, inter-patient variability in biomaterial retention, and when repeat dosing is necessary.

**Fig. 2:**
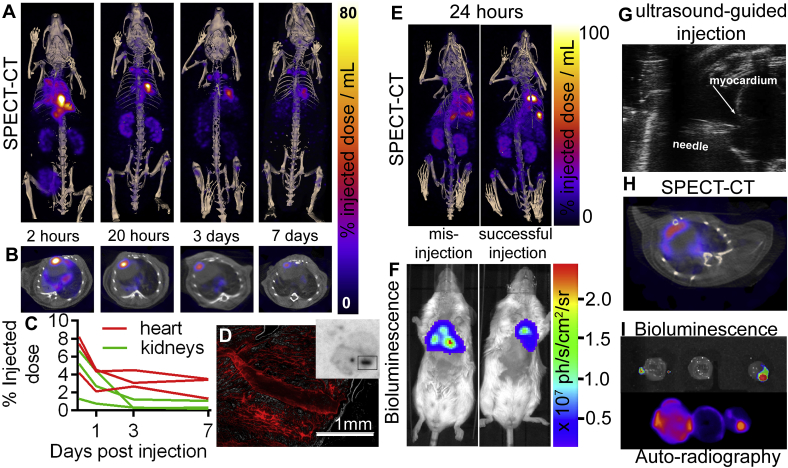
**A.** Maximum intensity projection SPECT image time-course showing retention of cardiac ^111^In alginate implantation and excretion of non-retained material *via* the kidneys and bladder **B.** Axial SPECT-CT sections show myocardial ^111^In-alginate co-localisation. **C.** SPECT-CT ROI activity quantification shows longitudinal kinetics of ^111^In-alginate cardiac retention and excretion via the kidneys in 3 separate animals following intracardiac injection at 0 h. **D.** Fluorescence microscopy of 20 μm sectioned heart tissue 10 days post injection showing TRITC-alginate the injection tract, with corresponding injection tract visible on the autoradiography image of the whole section. E. SPECT-CT of ^111^In-alginate + MSCs at 24 h following successful and unsuccessful injection, together with corresponding **F.** Bioluminescence images from the same mice confirming the location of live cells. **G.** Ultrasound-guided myocardial injection of ^111^In-labelled alginate. **H.** Axial SPECT-CT section showing co-localisation of ^111^In-alginate-MSCs SPECT signal with CT heart **I.***Ex vivo* bioluminescence showing live MSCs co-localises with ^111^In alginate at sites of implantation in corresponding *ex vivo* autoradiography of apical, mid and basal heart tissue sections from the successfully injected mouse shown in A-D at 10 days post-injection.

Intra-myocardial injections of (2% w/v) alginate were performed in mice under ultrasound guidance (Fig. 2G), and imaged over a week (Fig. 2A–C). At 2 h post-injection (Fig. 2A–C) it can be seen that the majority of injected alginate is not retained in the heart, and is cleared from the body over the following days via the kidney and bladder, consistent with previous pharmacokinetic data [24]. As a control ^111^InCl_3_ without alginate was also injected intra-myocardially, which gave a wider initial distribution in the chest cavity, and much faster clearance (Fig. S8). Two symmetrically-arranged high signal regions were also seen above the heart at 20 h to 7 days post-injection of ^111^In-alginate (Fig. 2A), suggesting clearance of the hydrogel to the mediastinal lymph nodes, which was not seen after injection of ^111^InCl_3_ as a control. The proportion of the injected alginate retained in the heart one week after injection was 2–4%, with SPECT signal coinciding with the myocardium in CT (Fig. 2B). The low cardiac retention fraction of implanted material is likely due to myocardial contraction and wall thickness of mouse hearts compared to humans, meaning alginate was rapidly expelled from the tissues. Imaging also showed inter-animal variability between initial fraction of implanted material in the heart of between 4 and 8% (Fig. 2C), indicating a clear role for biomaterial fate tracking. This experiment was then repeated using alginate labelled with the red fluorescent dye TRITC to assess ^111^In co-localisation with the implanted alginate. *Ex vivo* autoradiography and fluorescence microscopy of sectioned tissue both showed signal in the injection tract at 4 (Figs. S9A–C) and 10 days post-injection (Fig. 2D, S9D-I), confirming ^111^In retention in the implanted alginate.

In addition to the use of alginate for its native regenerative properties in the heart, it has been shown to enhance retention of injected therapeutic mesenchymal stem cells (MSCs) [25]. To assess the interactions between encapsulated cells and hydrogels *in vivo*, 2 × 10^6^ luciferase-expressing MSCs were suspended in 50 μL of ^111^In-alginate and injected into mouse hearts as above at 3 apical sites. In cases of successful injection SPECT-CT and bioluminescence show good spatial correlation between ^111^In-alginate and live cells respectively; this is also seen following mis-injections into the left ventricular cavity or pericardial space, which resulted in more disperse signals (Fig. 2E and F). At 10 days post-implantation, hearts were removed, sectioned, and *ex vivo* bioluminescence and autoradiography was performed (Fig. 2I). This showed good spatial co-localisation between *in vivo* SPECT-CT (Fig. 2H), auto-radiography, and *ex vivo* bioluminescence (Fig. 2I). Results suggest that live donor cells remained within the ^111^In-alginate at all 3 injection sites for at least ten days after injection. This approach can be used to provide valuable *in vivo* information on delivery and retention of alginate-encapsulated cells. Previous reports claim less than 0.1% of injected stem cells typically form stable grafts in the heart [26]. However, the use of alginate can enhance donor cell retention, attachment, and growth conditions, providing a rationale for both cellular and acellular use in cardiac repair [27].

## 4 Muscle implantation

**Fig. 3:**
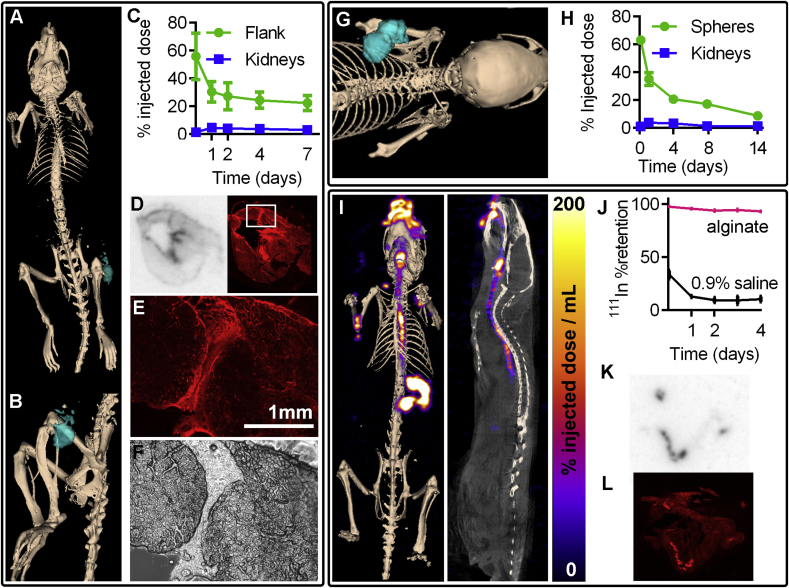
**SPECT-CT imaging and quantification of**^**111**^**In-alginate signal in models of muscle tissue engineering, stem cell grafting, and oral drug delivery. (A,B)** SPECT-CT 3D rendering showing ^111^In-alginate location (2 day post injection) in the flank muscle, and **(C)** signal quantification following injection into the flank muscle (n = 3, error bars show SD). The 0 day time point is 90 min post implantation **(D)** Autoradiography and matched fluorescence image of flank muscle 5 days post implantation showing co-localisation of TRITC-alginate and ^111^In signal. **(E)** Fluorescence and **(F)** brightfield microscopy images showing TRITC-alginate in the injection tract area of the thigh muscle highlighted in the box in part D. **(G)** SPECT-CT 3D rendering of ^111^In-alginate hydrogel spheres at 24-h post implantation and **(H)** quantification of signal up to two weeks after subcutaneous implantation. Points represent means (n = 3), error bars show SD. The 0 day time point is 90 min post implantation **(I)** Maximum intensity projection SPECT-CT and sagittal section showing ^111^In-Gaviscon (10% w/v alginate) in the oesophagus and stomach at 1 h post oral dosing. **(J)** Retention of ^111^In in Gaviscon and control saline solutions dialysed against simulated stomach acid (pH2). (**K**) Autoradiography and **(L)** fluorescence image of small intestine (20 μm section) 75 min post-dosing, showing co-localisation of alginate and ^111^In signal.

The ability to follow alginate in muscle is important for many emerging clinical therapies including bladder wall muscle chondrocyte implantation for vesicoureteral reflux treatment (Table S2), and regeneration of skeletal muscle diseases including muscular dystrophy [28]. As a model for muscle implantation 2% (w/v) ^111^In-alginate solution was injected into the thigh muscle of mice, and retention was followed over a week using SPECT-CT (Fig. 3A–C, S10A,B). The proportion of material successfully implanted and retained was much higher than the cardiac model, with over 20% retained a week post-injection (Fig. 3C). Clearance of free ^111^InCl_3_ injected in the same way as a control was faster than ^111^In-alginate (Fig. S11). Using fluorescently-labelled TRITC-alginate, we confirmed the co-localisation of ^111^In signal and alginate *ex vivo* on cryosections from tissue removed 5 days post-implantation. Autoradiography and fluorescence imaging showed overlapping areas of signal (Fig. 3 and S12) which could be clearly identified as the injection tract with fluorescence microscopy (Fig. 3E–F).

We then investigated ^111^In-alginate use as a support matrix for the differentiation of mesenchymal stem cells into chondrocytes *in vivo* (Fig. 3G,H, S10C,D, and Fig. S13). MSCs were suspended in ^111^In-alginate (2% w/v) cross-linked to form solid spheres in a bath of 100 mM CaCl_2_, before subcutaneous implantation. Alginate placement and degradation was then monitored up to 2 weeks using SPECT-CT, showing comparable retention of material to the flank-injected alginate. After 3 weeks the transplanted cell mass was removed and analysed histologically for the differentiation of stem cells into chondrocytes (Fig. S13). Cells in ^111^In-alginate retained their ability to differentiate into cartilage-like tissue, consistent with previous reports using non-labelled alginate [4]. MSCs grown in ^111^In-Alginate spheres also showed no change in cell growth compared to those grown in non-radiolabelled alginate (Fig. S14). This is consistent with the literature which shows that alginate is not taken up by cells unless their membranes are deliberately permeabilised [29], and the effective dose received by cells from ^111^In is several orders of magnitude lower when the isotope is outside the cell than within it [30].

As various alginate-encapsulated cell types are being investigated for therapies, including Islets of Langerhans for diabetes [31], and hepatocytes for acute liver failure [32], the ability to image transplanted alginate beads as presented here could be of wide utility in understanding their behaviour *in vivo* and potential routes for optimising transplantation strategies.

## 5 Oral drug delivery

Alginate is also widely used to treat gastro-oesophageal reflux disease (GORD, also GERD, or acid reflux), which affects 40% of the Western population [33]. In addition to heartburn, GORD increases risk of oesophageal adenocarcinoma by 8-fold [34]. Orally-dosed alginate solutions are the best-established treatment for GORD, acting as a mechanical floating barrier to reflux [33]. The large market size for GORD treatments creates substantial pharmaceutical research interest in optimising alginate-based formulations for retention in the stomach [33,35] which is necessary for efficacy but difficult to monitor non-invasively. We labelled an over-the-counter alginate-based anti-acid formulation Gaviscon™ (containing 10% w/v alginate) with ^111^In, and measured ^111^In retention following dialysis against simulated stomach acid (pH 2 Krebs-Henseleit buffer + 1 mM deoxytaurocholate), which showed good retention up to 4 days (Fig. 3J). We then dosed this to mice and imaged them up to 4.5 h post-dosing with SPECT-CT. Retention in the stomach was quantified, giving: 44.8% retention at 20 min post dosing, 43.9% at 1.5 h, and 37.5% at 4.5 h (Fig. 3 and S15). For *ex vivo* validation ^111^In Gaviscon was mixed with TRITC-alginate and dosed as above. Autoradiography and fluorescence imaging of tissue sections showed retention of ^111^In-alginate in the small intestine, confirming persistent binding (Fig. 3K,L, and S16). This shows that non-invasive *in vivo* imaging was suitable for assessing delivery kinetics of this commercial formulation. This ability to image orally-delivered alginate will also be useful for the development of for other emerging oral uses including drug delivery for diabetes mellitus [36] and (re)population of beneficial microbiota in the gut [37].

## 6 Nasal drug delivery to brain

Finally, we showed that alginate could be tracked in mice after nasal dosing, where alginate's mucoadhesive properties and viscosity suit its use as a vehicle. This is a promising route for delivering small or macro-molecules to the brain as it bypasses the blood brain barrier [38]. Past studies have shown greater retention of viscous compared to non-viscous fluids in the nose, however multiple interacting parameters such as spray device and droplet size make delivery unpredictable, providing a valuable role for imaging in quantifying delivery and retention [39,40].

**Fig. 4:**
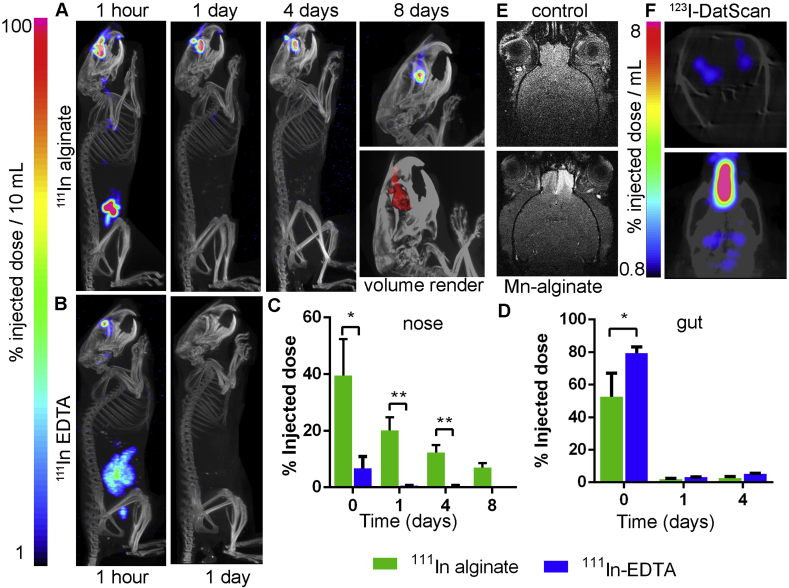
^111^In-Alginate shows enhanced retention in the nose compared to a control solution of ^111^In-EDTA. Maximum intensity projection SPECT-CT image timecourse showing retention of **a.**^111^In-Alginate and **b.**^111^In-EDTA after nasal administration. **c.** A significantly higher % ID is retained in the nose in the alginate group compared to control up to 4 days (n = 3; 2-tailed T-test; *p < 0.01, **p < 0.001). **d.** A higher % of the injected dose is present in the abdomen in the control group, showing gut clearance, at 90 min after dosing (0 days), vs the alginate group (n = 3; 2-tailed T-test; *p < 0.05). Error bars show SEM. **e.** Axial T_1_-weighted magnetic resonance image showing signal hyper-intensity in the olfactory bulb after nose-to-brain delivery of Mn^2+^ from alginate, but not in the control condition before dosing. **f.** Coronal section and dorsal maximum intensity projection SPECT-CT images showing signal in the basal ganglia following nasal delivery of ^123^I-DatScan in 0.6% alginate, confirming nose-to-brain delivery to pre-synaptic dopamine transporters within 2 h of dosing.

We compared nasal retention of a low-viscosity control aqueous solution containing a soluble indium chelate (^111^In-EDTA) to an ^111^In-cross-linked 0.6% (w/v) alginate solution. Mice were dosed with 20 μL of solution into each nostril *via* pipette. SPECT-CT imaging showed significantly higher retention of ^111^In-alginate vs ^111^In-EDTA, with a 5.8-fold higher activity retention at 1 h, 35-fold at 24 h and 26-fold at day 4.^111^In-alginate was still detectable at days 8 and 14 post-dosing, whereas no signal remained in the animals receiving ^111^In-EDTA (Fig. 4A–C). Gut signal was consistent with these results, showing significantly more clearance via this route in the mice receiving the ^111^In-EDTA compared to ^111^In-alginate (Fig. 4D).

To confirm that nasally-dosed alginate can transfer its cargo to the brain, hydrogels were loaded with the MRI contrast Mn^2+^ (0.6% w/v alginate, 10 mM MnCl_2_) and dosed before imaging 30 min later using MRI (Fig. 4 e). Mn^2+^ in contrast to Ca^2+^, In^3+^ and Zr^4+^, binds poorly to alginate and is quickly released [41]. Signal hyper-intensity of the olfactory bulb was seen following Mn-alginate dosing but not in the control condition (lacking Mn^2+^), confirming nose-to-brain delivery. To demonstrate delivery of a more typically drug-sized small molecule to the brain, ^123^I-Datscan (MW = 427.285 g/mol) was dosed as above in 0.6% w/v alginate and detected using SPECT. Uptake of ^123^I-DatScan in the basal ganglia (Fig. 4F) where it binds to presynaptic dopamine transporters, confirms its ability to reach distant brain target regions following nasal dosing.

Other clinical applications of nasal alginate delivery include post-surgical application to prevent bleeding [42], with dust mite extracts for hypo-sensitisation in rhinitis patients [43], and for controlled drug release in the nose [44].

## 7 Discussion

This report extends the recent progress on chelate-free radio-labelling beyond its current domain of nano-materials (including: iron oxide nanoparticles [[45], [46], [47]], nanophosphors [48], gold nanoparticles [49], liposomes [50], silicon nanoparticles [51], and nanographene [52]) into the field of polymers and hydrogels. The simple and novel radio-metal cross-linking technique we describe here uses the natural ability of alginate to co-ordinate multivalent cations in place of the more traditional chelate-based radiolabelling approaches which require additional chemical modification. We have shown that alginate sugar subunits bind In^3+^ ions in a similar way to the cross-linker Ca^2+^ which is routinely used in biomedical applications. This allows the location of alginate hydrogels or solutions to be measured across the anatomy in three dimensions using non-invasive SPECT imaging. We have shown that radio-metal incorporation does not alter mechanical hydrogel properties and remains bound within the biomaterial to enable *in vivo* imaging over biologically-relevant timescales. Though ^89^Zr did not turn out to be well-suited to this labelling technique, its potential compatibility with other radio-metal cations such as ^64^Cu^2+^, ^68^Ga^3+^, and ^177^Lu^3+^ for PET and SPECT imaging, as well as radiotherapy, is the source of ongoing investigation.

We have demonstrated the versatility of this technique in a number of mouse models of pre-clinical alginate-based interventions, showing the *in vivo* biodistribution of alginate used for acellular and cellular tissue engineering in the heart and muscle, stem cell grafting, and oral and nasal drug delivery. The information thus acquired informs on the success of delivery or transplantation in the target tissue, its retention over time, clearance, off-target accumulation, and inter-patient/inter-animal and inter-dose variability. This will be invaluable data during the evaluation of novel alginate-based therapies in preclinical development and clinical trials (Table S2). It will provide better understanding of the safety of novel treatments, their mechanisms of action, and feedback for optimisation of formulations and dosing strategies. This technique uses materials (alginate, ^111^In) and quantitative imaging (SPECT) already available in the clinic, lowering the barrier to implementation.

Though previous methods have been described to image alginate hydrogels with fluorescence, MRI, CT, and ultrasound [[53], [54], [55]], these imaging modalities variously suffer from ambiguities of detection which are not encountered with the nuclear imaging systems that we describe here. Fluorescence imaging is comparatively cheap and widely-available, but suffers from poor depth penetration and quantification, unlike nuclear imaging. MRI has the advantage of better tissue depth penetration than fluorescence, suiting it better for clinical application and whole body imaging, while also allowing higher resolution detection of labelled material than nuclear imaging. But it is not always possible to unambiguously detect contrast agents such as iron oxides with MRI in certain areas of the body in which there is endogenous heterogeneity in signal or hypo-intense signal voids. For example any gas filled cavity such as the nasal cavity, oesophagus, lungs, or bowels, has its own susceptibility artefacts which prevent quantification. With CT, endogenous contrast is provided by the bones and soft tissue, and the mass of contrast agent needed to be incorporated into the hydrogel to provide signal is up to seven orders of magnitude greater than it is for nuclear imaging [56]. This may be a problem because introducing much larger amounts of contrast agent is likely to alter the material properties of the hydrogel, and influence the behaviour of encapsulated drugs or cells, unlike the (sub)nanomolar amounts of radio-metal required for SPECT or PET. Though ultrasound imaging is convenient and cost effective for certain applications, the areas of the body that can be imaged are again limited, and signal impeded by bone or pockets of gas - preventing quantification of material distribution across the whole body. On the other hand, longitudinal SPECT-CT allows signal quantification across the entire anatomy, with its use mainly restricted by the chosen isotopes’ half-life, which limits practical detection to the order of one or two weeks in the case of ^111^In. In terms of safety, ^111^In is typically used for diagnostic purposes in the clinic at doses between 12 and 220 MBq per patient [57,58]. Thus it would be realistic to assume that doses several-fold higher than those used in this study (5–6 MBq) could be justified to reduce imaging time or extend the time-course of material tracking beyond the week to a fortnight demonstrated here.

In summary, we have demonstrated for the first time that alginate can be cross-linked with a radio-metal (^111^In), and that this enables imaging of its *in vivo* biodistribution with SPECT-CT. Nuclear imaging allows three-dimensional non-invasive *in vivo* imaging with good resolution across the anatomy, and is available for both pre-clinical and clinical use. Due to the versatility of alginate as a biomaterial and the prevalence of novel emerging uses in drug delivery and regenerative medicine, we anticipate that this labelling method will be of value to the biomaterial community in the development, optimisation, and validation of novel alginate-based therapies.

## 8 Experimental section

Experimental details are provided in greater detail the supporting information.

## 9 Material characterisation

XPS and ATR-FTIR were performed on hydrogel beads made using 3% w/v sodium alginate solution drop-cast into aqueous 300 mM InCl_3_ and ZrCl_4_ solutions and left to cross-link for 10 min. These were then incubating for 30 min in distilled water three times to wash off unbound metal salts, before freeze-drying overnight.

X-ray Photoelectron Spectroscopy (XPS) was performed using a Thermo Scientific K-alpha spectrometer with monochromated Al Kα radiation, a dual beam charge compensation system and constant pass energy of 50 eV (spot size 400 μm). High-resolution peaks were used for the principal peaks of C (1s), Na (1s), Ba (3d), Ca (2p), In (3d) and Zr (3d). Peaks were modelled with CASA XPS software.

Attenuated Transmission Reflectance Fourier-Transform Infrared (ATR-FTIR) spectra were recorded on a Bruker ALPHA Platinum ATR single reflection diamond ATR between 400 cm^−1^ and 4000 cm^−1^.

## 10 SEM

Alginate beads were prepared for SEM using a drop casting method with a 3% sodium alginate solution (w/v) and 300 mM CaCl_2_ solution as cross-linker. Relevant molar equivalents of ZrCl_4_ or InCl_3_ were added to the alginate solution prior to cross-linking with calcium, assuming specific activities of 0.15 GBq/nmol and 1.85 GBq/nmol respectively. Samples were changed through an ethanol series then stored in a fresh solution of 100% ethanol before drying in a Quorum K850 critical point dryer. Samples were then fractured under liquid nitrogen, and given a 5 nm gold coating in a Quorum Q150T Sputter coater. Images were obtained at 50 000× and 70 000× magnification using a FEI Quanta 200 FEG ESEM (ThermoFisher Scientific).

## 11 Viscosity measurements

Viscosity measurements were made on 1 mL samples over a shear rate range of 90–1000 s^−1^ using a Bohlin CVO rheometer (Malvern Instruments). This was set up in the cone and plate geometry (60 mm, 0.995°), and the temperature maintained at 20 ± 0.05°. A gap of 25 μm was used for all experiments.

## 12 Radioactivity measurements

All radioactivity measurements were made using either a dose calibrator (Capintec Curimentor), or where appropriate an automated high-sensitivity gamma counter (Perkin Elmer Wizard^2^). For *in vivo* analysis of activity, regions of interest were drawn manually around sites of injection using the CT data for anatomical reference, using VivoQuant software (Invicro).

## 13 Cell culture

Luciferase-expressing D1 ORL UVA (ATCC® CRL-12424™) mouse mesenchymal stem cells (a kind gift from Dr Arthur Taylor, University of Liverpool) were grown in DMEM (Invitrogen, UK) including 10% fetal bovine serum (FBS), 2 mM l-glutamine and 0.1 mM MEM nonessential amino acids at 37 °C with 5% CO_2_ and 95% humidity.

Cell viability was measured as photon output 6 min after the addition of luciferin to the growth media (final concentration of 50 μg/mL), using an optical plate-reader (Varioskan Lux, Thermo-Fisher). Growth media was changed following each assay reading. A total of 11 alginate beads were synthesised for each condition by suspending MSCs at a concentration of 20 million per mL in a 1.5% (w/v) alginate solution. Cross-linking was done for 10 min using a 100 mM CaCl_2_ solution, with a bead volume of 100 μL. Beads were cultured in individual wells of an opaque cell culture dish. For the Indium condition, ^111^InCl_3_ was added to the alginate solution at 30 MBq per mL final concentration.

## 14 Autoradiography

For co-registration with fluorescence images, excised hearts, muscles, and intestines were frozen in Optimal Cutting Temperature solution (OCT; Cellpath), and sectioned at 20 μm using a cryotome (Leica CM3050S) onto slides (SuperFrost; ThermoScientific). Fluorescence microscopy was done using an EVOS XL (Thermofisher) equipped with RFP filters (Ex. 531 nm, Em. 593 nm), and whole slide fluorescence images were obtained at 50 μm resolution (Ex. 532 nm, Em. 580) using a Typhoon 9410 Imager (GE Healthcare). The same slides were then exposed to a storage phosphor screen (GE Healthcare) for between 10 min and 3 days to obtain sufficient signal, which was scanned at 50 μm resolution (Typhoon 9410 Imager), and the resulting images were processed by ImageJ (NIH, USA).

For co-registration with *ex vivo* bioluminescence, hearts were excised, flash frozen, and cut into 1 mm thin sections along the short axis, before incubation with 1% TTC for 30 min. Sections were mounted on, photographed and exposed to GP 20 × 25 cm^2^ phosphor screens (VWR international LTD, U.K.) for 2 h. After this time, the screens were scanned by a Typhoon 9410 Trio + Phosphorimager (GE Healthcare, U.K.), (25 μm resolution, acquisition time = 2 h 30 min) and the resulting images were processed by ImageJ (NIH, USA).

## 15 Alginate fluorescent labelling

Alginate was labelled with tetramethylrhodamine (TRITC; Excitation maximum = 543 nm; Emission maximum = 569 nm) using an EDC/NHSS conjugation protocol adapted from Zhu et al. [59]. To confirm labelling, the fluorescence spectrum of the labelled alginate was then measured using a microplate reader (VarioSkan Lux; ThermoFisher) with comparison to a TRITC dye solution and unlabelled alginate (Fig. S17).

## 16 Histology

Alginate spheres were excised at 20 days post implantations, at which point teratoma formation had occurred. Tissues were fixed overnight in 4% paraformaldehyde then changed into 70% ethanol. Samples were dehydrated and paraffin embedded before sectioning at 20 μm thickness onto glass slides and stained with Alican blue, and counterstained with nuclear fast red solution (Sigma Aldrich product: N3020-100 ML).

## 17 In vivo animal work

All animal studies were approved by the University College London Biological Services Ethical Review Committee and licensed under the UK Home Office regulations and the Guidance for the Operation of Animals (Scientific Procedures) Act 1986 (Home Office, London, United Kingdom). All animal methods were performed in accordance to institutional ethical guidelines and regulations. C57BL/6 animals were all acquired from Charles River.

For intramyocardial delivery, 10 week old, male C57BL/6 mice (n = 3) were anaesthetised with 2% isoflurane in 100% O_2_. Hearts were visualised using ultrasound (Visualsonics Vevo 2100) and 50 μL of 2% ^111^In alginate with or without MSCs (4 × 10^7^ cells/mL alginate) was injected into the anterolateral wall of the myocardium using ultrasound guidance of a 30 gauge needle. Ten days later, hearts were flushed with saline, excised and sliced for autoradiography and *ex vivo* BLI.

For muscle delivery, 10 week old, male C57BL/6 mice (n = 3) were anaesthetised with 2% isoflurane in 100% O_2,_ and 50 μL of 2% ^111^In-alginate was injected into the left thigh muscle using a 30 gauge needle.

For subcutaneous bead implantation, 2 mm diameter, ^111^In alginate beads were fabricated by dropcasting of 2% (w/v) alginate pre-mixed to a final concentration of 2 × 10^7^mouse mesenchymal stem cells/mL into a bath of 100 mM CaCl_2_ and left to cross link for 4 min 10 week old, male C57BL/6 mice (n = 3) were anaesthetised with 2% isoflurane in 100% O2, Fur was removed from the dorsal region, the skin was sterilised and a small incision was made between the shoulder blades. Beads (6–7 beads per animal) were implanted into the subcutaneous space and the incision was closed with a suture.

For oral administration of ^111^In-labelled anti-acid treatment, Gaviscon™ Advance Original Aniseed (Boots Plc, Tottenham Court Road, England) was mixed with ^111^InCl_3_ at a ratio of 5 MBq per 100 μL of Gaviscon. 10 week old, male C57BL/6 mice (n = 2) were scruffed and voluntarily consumed the ^111^In-Gaviscon (100 μL) which was dispensed from a pipette tip positioned next to the mouth. Mice were then imaged with SPECT-CT at 20 min, 90 min, and 4.5 h (270 min) after administration.

For nasal dosing of ^111^In-alginate, a solution was prepared to a final concentration of 0.6% w/v alginate containing 150 MBq/mL ^111^InCl_3_. Of this, 20 μL was administered to each of the left and right nasal passages using a micropipette (Gilson) while the animal was scruffed, resulting in doses of ~6 MBq ^111^In per animal. 10 week old, male C57BL/6 mice (n = 3) were used per condition. As a control, mice were administed an equal volume of ^111^In-EDTA (Ethylenediaminetetraacetic acid; Sigma Aldrich) which was made prepared by mixing a 1 mM EDTA solution (pH 7.4) with ^111^InCl_3_ to a final activity of 150 MBq/mL. Thin layer chromatography using a 0.9% saline mobile phase was used to confirm binding of ^111^In to EDTA before administration; unbound ^111^In will remain at the origin while ^111^In-EDTA will migrate with the solvent front. For nasal dosing of 0.6% alginate containing ^123^I-DatScan (20 MBq/100 μL), 20 μL was dosed per nostril using a micropipette and SPECT-CT imaging was performed at 2 h post-dosing.

For MRI nasal dosing of Mn-alginate was done using a micropipette to administer 20 μL of a 0.6% solution of alginate (w/v) containing 10 mM MnCl_2_. Imaging was performed at 30 min post dosing.

## 18 In vivo imaging

During all *in vivo* imaging, mice were maintained at 37 °C under isofluorane breathable anaesthesia (1–2%) in oxygen. A small animal physiological monitoring system (SA Instruments, Stony Brook, NY) was used to monitor core body temperature and respiration rate.

SPECT data was acquired using a NanoScan SPECT-CT (Mediso) with 1.4 mm pinholes, interfaced to a computer running Interview Fusion software (Bartec). Images were reconstructed using HiSPECT software, and analysed using VivoQuant software (Invicro). CT images were acquired using a 55 kVP X-ray source, 500 ms exposure time, 180 projections, a pitch of 1.5. CT images were reconstructed in voxel size 124 × 124 × 124 μm, whereas SPECT images were reconstructed in a 256 × 256 matrix prior to being overlayed. 3D ROIs were manually drawn and used to calculate the percentage of injected dose/organ (%ID/organ).

PET data was acquired using a NanoScan PET-CT (Mediso) interfaced to a computer running Interview Fusion software (Bartec), which was also used to reconstruct images. Data was analysed using VivoQuant software (Invicro).

MRI data was acquired using a 9.4T horizontal bore scanner (Varian) interfaced to a VNMRJ 3.1 (Varian) imaging console, using an Agilent 205/120HD gradient set, 72 mm inner diameter volume coil for RF transmission (Rapid Biomedical), and a 2 channel array surface coil (Rapid Biomedical) for signal reception. T_1_-weighted images were acquired using a multi slice gradient echo (TE 2.5 ms/TR 75 ms; 75° flip angle), with a 100 × 100 × 250 μm voxel size.

*In vivo* Bioluminescence imaging was performed using an IVIS 200 (PerkinElmer) imaging device at 10 min post intraperitoneal injection of D-luciferin (Promega) solution at 150 mg/kg per mouse. *Ex vivo* imaging was performed immediately after cervical dislocation and dissection of the animal.

## Data availibility

Data will be made available on request.

## Author Contributions

PSP and DJS conceived the study, designed and performed experiments, and analysed data. JCB designed and performed experiments and analysed data. HEF and MZT performed experiments and analysed data. PSP wrote the paper. IPP, MFL, TLK, and DJS supervised the study and edited the paper.

## CRediT authorship contribution statement

**P. Stephen Patrick:** Conceptualization, Methodology, Formal analysis, Investigation, Supervision, Writing - original draft, Writing - review & editing, Visualization, Project administration, Funding acquisition. **Joseph C. Bear:** Methodology, Formal analysis, Investigation, Data curation, Writing - review & editing, Visualization. **Heather E. Fitzke:** Investigation, Writing - review & editing. **May Zaw-Thin:** Investigation, Writing - review & editing. **Ivan P. Parkin:** Supervision, Writing - review & editing. **Mark F. Lythgoe:** Supervision, Writing - review & editing. **Tammy L. Kalber:** Investigation, Writing - review & editing. **Daniel J. Stuckey:** Conceptualization, Methodology, Investigation, Supervision, Writing - review & editing, Funding acquisition.

## Declaration of competing interest

The authors declare that they have no known competing financial interests or personal relationships that could have appeared to influence the work reported in this paper.

The authors have no relevant affiliations or financial involvement with any organization or entity with a financial interest in or financial conflict with the subject matter or materials discussed in the manuscript apart from those disclosed.
