# Supplementary material for: Radio-metal cross-linking of alginate hydrogels for non-invasive in vivo imaging
Source: Biomaterials. 2020 Jun;243:119930. doi: 10.1016/j.biomaterials.2020.119930 (PMC7103761; doi:10.1016/j.biomaterials.2020.119930)
Supplement: Multimedia component 1 [file mmc1.docx]

**Supplementary Information**

**Radio-metal Cross-linking of Alginate Hydrogels for Non-invasive in vivo Imaging**

P. Stephen Patrick*, Joseph C. Bear, Heather E. Fitzke, May Zaw-Thin, Ivan P. Parkin, Mark F. Lythgoe, Tammy L. Kalber, and Daniel J. Stuckey*

*P. Stephen Patrick, Heather E. Fitzke, May Zaw-Thin, Mark F. Lythgoe, Tammy L. Kalber, and Daniel J. Stuckey- Centre for Advanced Biomedical Imaging, University College London, London WC1E 6DD, UK.*

*Joseph C. Bear –* School of Life Science, Pharmacy & Chemistry, Kingston University, Penrhyn Road, Kingston upon Thames, UK

*Ivan P. Parkin- Materials Chemistry Centre, Department of Chemistry, University College London, 20 Gordon Street, London, WC1H 0AJ, UK.*

**Joint corresponding authors***

*P. Stephen Patrick.* peter.patrick@ucl.ac.uk

*Daniel J. Stuckey.* d.stuckey@ucl.ac.uk

*Centre for Advanced Biomedical Imaging*

*University College London,*

*London*

*WC1E 6DD,*

*UK.*

***Table of Contents:***

Table S1 - Selection of commercial alginate based medical products.

Table S2 - Selected Alginate-based clinical trials listed on clinicaltrials.gov.

Table S3. C1s area ratios

Table S4 ATR-FTIR peaks showing COO^-^ stretching

Table S5 ATR-FTIR peaks showing C-O stretching

Figure S1 – XPS spectra showing metal ion contents

Figure S2 - XPS spectra showing carbon binding energies

Figure S3: ATR-FTIR spectra of the metal alginate complexes

Figure S4. Radiolabel retention following dialysis

Figure S5 SEMs of In and Zr labelled alginate hydrogels

Figure S6 Rheometry measurements following In and Zr labelling

Figure S7 ^89^Zr-alginate PET imaging following myocardial injection

Figure S8 SPECT imaging of ^111^In-alginate in thigh muscle and as subcutaneous beads

Figure S9 Histology showing chondrogenesis of MSCs implanted in ^111^In-alginate beads

Figure S10 MSC growth in cross-linked (1% w/v) alginate beads with or without ^111^In.

Figure S11 SPECT-CT images showing ^111^In-Gaviscon biodistribution after oral delivery.

**Table S1. Selection of commercial alginate based medical products.**

| **Disease area** | **Alginate form** | **Alginate use** | **Product name / sponsor** | **Manufacturer** |
| --- | --- | --- | --- | --- |
| Peridontal | Propylene Glycol Alginate and porcine Enamel Matrix Derivative | Tissue engineering- promotion of regrowth in hard and soft periodontal tissues | Emdogain ^R^ | Straumann BG |
| Dental | Alginate hydrocolloid | Preparation of dental impressions | Many products incl. | Zhermack, Lascod |
| General post-surgery barrier application | Poloxamer and Sodium Alginate (injectable or applied via spray) | Tissue engineering | Guardix-SG ^R^ | Hanmi Pharm. Co. Ltd. |
| Wounds | Calcium alginate, arboxymethylcellulose, silver sodium hydrogen zirconium phosphate | Antibiotic wound treatment | Tegaderm ^TM^ | 3M ^TM^ |
| Wounds | Translucent alginate gel | Wound healing | Aquaflo ^TM^  Curafil ^TM^ | Covidien |
| Wounds | Multilayer composite with Alginate hydrocolloid | Wound healing | Ultrec Pro ^TM^ | Kendall |

**Table S2. Alginate-based clinical trials listed on clinicaltrials.gov.** Alginate is being used wide range of disease areas, including as a cell scaffold, an acellular structural component for tissue engineering, for delivery of small molecule drugs and probiotics

| **Disease area** | **Alginate form** | **Alginate use** | **Cell type** | **Product name / sponsor** | **Trial Phase** | **Clinical trials.gov ID** |
| --- | --- | --- | --- | --- | --- | --- |
| Parkinson's Disease | Alginate capsule | Cell scaffold material | Porcine Choroid plexus cells | NTCELL/  Living Cell Technologies | I / II  IIb | NCT01734733  NCT02683629 |
| Stroke With Space-occupying Intracerebral Haemorrhage | Alginate beads | Cell scaffold material | allogenic mesenchymal cells, transfected to secrete Glucagon like peptide-1 | GLP-1 CellBeads® /  CellMed AG | I / II | NCT01298830 |
| Vesicoureteral Reflux | Gel suspension | Cell scaffold material | Chondrocytes | FDA Office of Orphan Products Development | III | NCT00004487 |
| Type I Diabetes | Alginate capsule | Cell scaffold material / immune-protection | Porcine Islets | DIABECELL /  Living Cell Technologies | I / IIa  I / IIa  IIb | NCT00940173  NCT01739829  NCT01736228 |
| Type I Diabetes | Alginate monolayer | Cell scaffold material | Allogenic Human Islets | Cliniques universitaires Saint-Luc- Université Catholique de Louvain | I | NCT00790257 |
| Introduction of microbiota post-operation | Sodium alginate | Oral bacteria delivery | L. rhamnosus, L. jensenii, L. crispatus, L. gasseri | Medical University of Vienna | N/A | NCT01708148  NCT01723592 |
| Constipation-predominant irritable bowel syndrome | Starch-entrapped alginate microspheres | Drug delivery, promotion of gut microbiota | N/A | Nutrabiotix fiber / Nutrabiotix, LLC | I / II  II | NCT01210625  NCT02144753 |
| Cystic fibrosis | Nebulised alginate solution  / dry powder | Drug delivery (inhalation) | N/A | Oligo G / AlgiPharma AS | IIb  II | NCT02157922  NCT02453789 |
| Pulmonary air leakage | Alginate hydrogel sleaves | Surgical reinforcement | N/A | FORESEAL/ Hôpitaux de Paris | IV | NCT00925444 |
| Heart failure | Injectable Alginate hydrogel | Tissue engineering - Left Ventricular Augmentation | N/A | Algisyl-LVR /  LoneStar Heart, Inc. | II / III | NCT01311791 |
| Myocardial infarction | Sodium Alginate and Calcium Gluconate | Tissue engineering - Remodeling of the Ventricle | N/A | IK-5001 /  Bellerophon BCM LLC | I | NCT01226563 |

**X-ray Photo-electron Spectroscopy (XPS):**

XPS spectra for sodium, barium, calcium, indium and zirconium represented a number of challenges, not least the fitting of the C1s spectra. Incorporation of different metal ions changed the modes of interaction in the alginate structure when compared to the native sodium. Carbon within the alginate structure is always bonded to at least one oxygen, so calibration of the main/any C1s peaks to adventitious (C-C) carbon proved impossible. For all cations, a model of 4 chemical environments was fitted, corresponding to C-C, C-O, O-C-O and COOH in order of increasing binding energy. 4 environments identified in C1s corresponding to C-C, C-O, O-C-O and COOH groups. ^[1]^ The areas of C1s fitted environments were also compared revealing different bonding modes favoured by different metal ions. In particular, COOH environment became more prevalent with increasing cationic charge, and the coordination of ethereal groups decreased with multi-valent cation incorporation, indicating bonding preferences. A rigorous investigation into uptake of metal ions by alginate gels taking into account metal ion concentration is ongoing.

The metal ion peaks were also examined by high resolution XPS, indicating Na^+^, Ba^2+^, Ca^2+^, In^3+^ and Zr^4+^.

Sodium alginate:

Na1s = 1071.60 eV good match for sodium/organic salts ^[2]^ - 4 environments identified in C1s corresponding to C-C, C-O, O-C-O and COOH groups. ^[1]^ - B.E. peaks at 285.00, 286.58, 288.18, 289.28 eV.

Barium alginate:

Ba3d: DS = 3.50 eV ^[3]^. Ba(II)- B.E. peaks at 780.28 eV and 795.58 eV. ^[4]^ 4 environments identified in C1s corresponding to C-C, C-O, O-C-O and COOH groups. ^[1]^ - B.E. peaks at 284.88, 286.38, 287.69 and 289.11 eV.

Calcium alginate:

Ca2p: DS = 3.50 eV ^[5]^. Ca(II)- B.E. peaks at 347.42 and 350.98 eV. ^[6]^ - 4 environments identified in C1s corresponding to C-C, C-O, O-C-O and COOH groups. ^[1]^ B.E. peaks at 284.76, 286.40, 287.59 and 288.60 eV.

Indium alginate:

In3d: DS = 7.60 eV ^[7]^ Indium(III)- B.E. peaks at 445.78 and 453.38 eV. Indium triiodide ^[8]^- 4 environments identified in C1s corresponding to C-C, C-O, O-C-O and COOH groups. ^[1]^ B.E. peaks at 285.03, 286.63, 287.95 and 289.04 eV.

Zirconium alginate:

Zr3d: DS = 3.50 eV ^[9]^. Zr(IV)- B.E. peaks at 182.98 and 185.28 eV. Zirconyl Chloride ^[10]^ - 4 environments identified in C1s corresponding to C-C, C-O, O-C-O and COOH groups. ^[1]^ B.E. peaks at 285.10, 286.53, 287.98 and 289.00 eV.

Table S3. C1s area ratios:

| Metal ion | Rel. area (C-C) / % conc. | Rel. area (C-O) / % conc. | Rel. area (O-C-O) / % | Rel. area (COOH) / % |
| --- | --- | --- | --- | --- |
| Na^+^ | 24.82 | 48.38 | 22.20 | 4.60 |
| Ba^2+^ | 61.03 | 25.30 | 9.74 | 3.93 |
| Ca^2+^ | 60.18 | 24.95 | 8.88 | 5.98 |
| In^3+^ | 35.16 | 42.55 | 12.46 | 9.83 |
| Zr^4+^ | 60.77 | 23.38 | 5.05 | 10.80 |


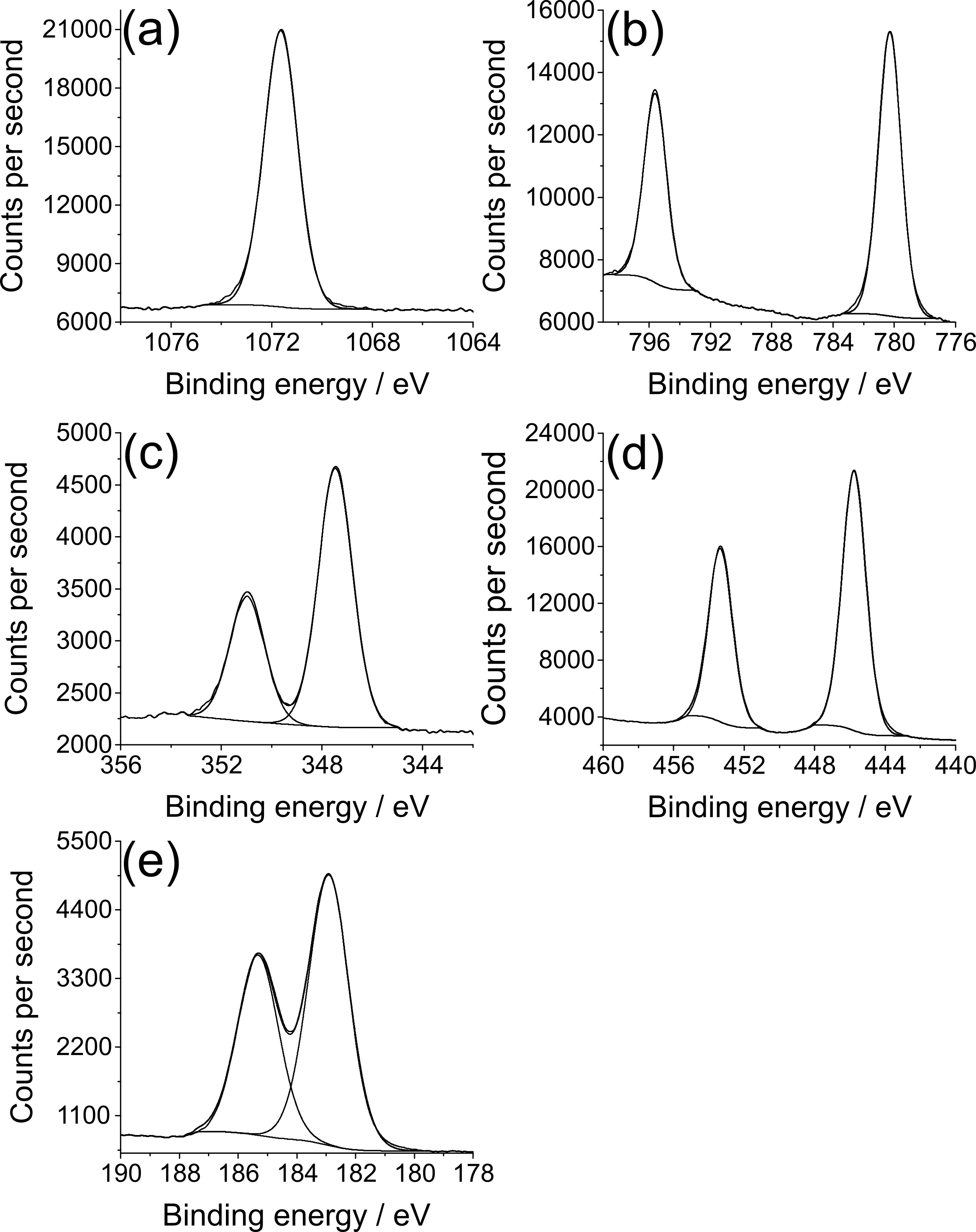


**Figure S1:** Fitted high resolution spectra of: (a) Sodium alginate (Na1s), (b) barium alginate (Ba3d), (c) calcium alginate (Ca2p), (d) indium alginate (In3d) and (e) zirconium alginate (Zr3d). All indicated desired oxidation states in single chemical environments, *i.e.* Na^+^, Ba^2+^, Ca^2+^, In^3+^ and Zr^4+^ from their respective binding energies.

**
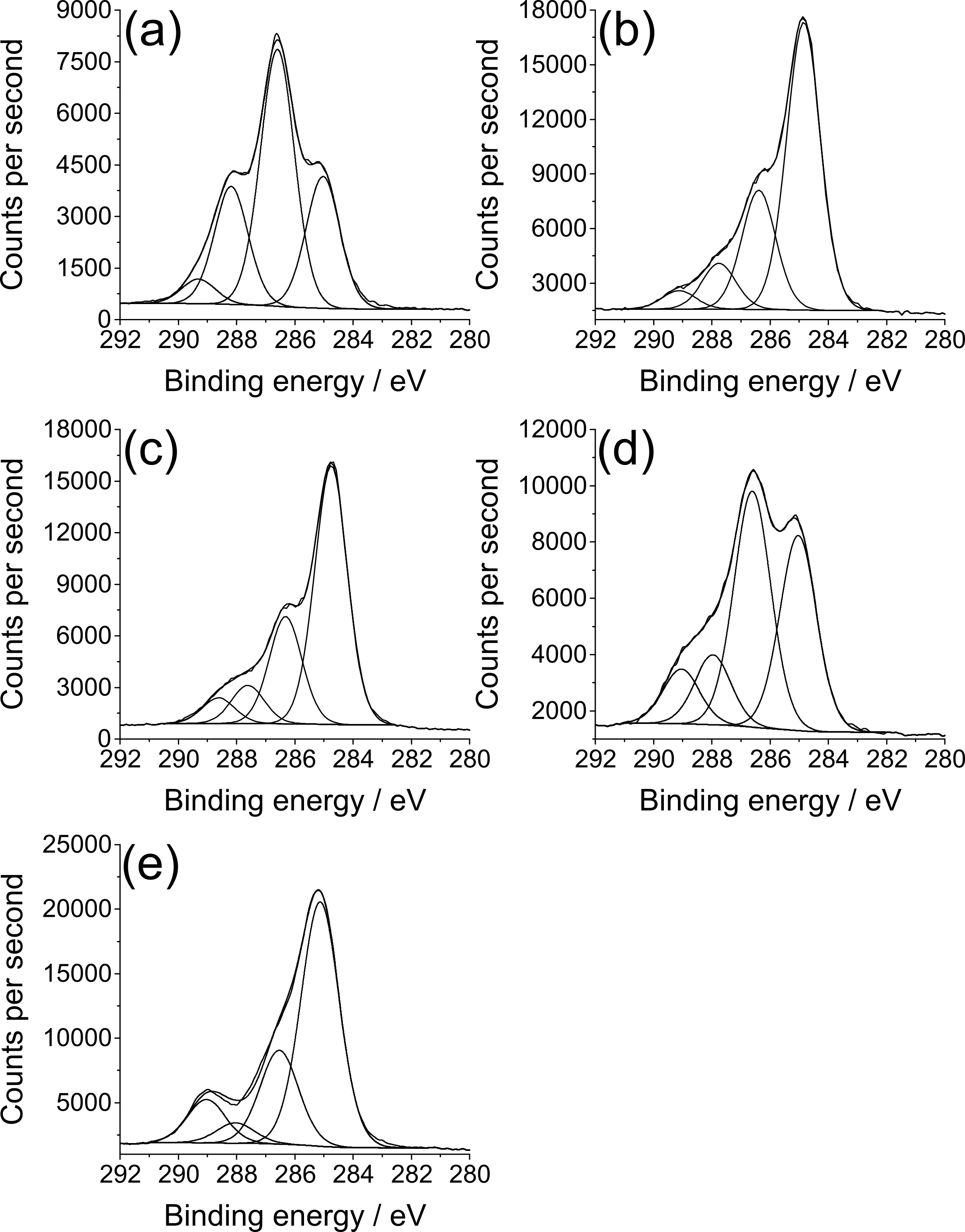
**

**Figure S2:** Fitted high resolution C1s spectra of: (a) Sodium alginate, (b) barium alginate, (c) calcium alginate, (d) indium alginate and (e) zirconium alginate.

**Attenuated Total Reflection – Fourier Transform Infrared Spectroscopy (ATR-FTIR) spectra**

When looking at the FTIR spectra of metal-alginate complexes, it is important to remember that differences in IR peak positions largely depend on ion concentration in the metal-alginate complex.

Sartori *et al.*^[11]^observed that the amount of metal ion binding to the alginate was the most important factor governing changes in the FTIR spectra, rather than simply the size or charge of metal ion. That being so, we can see that the changes in wavenumbers of stretching frequencies associated with carboxyl, hydroxyl and ethereal species in the metal alginate complexes have changed markedly on binding with each metal ion. Therefore we can say the metal has indeed cross-linked the alginate. Work determining the extent, and how different metal ions bond to the alginate as a function of concentration, ionic radius and charge is the focus of a rigorous ongoing study. In summary, shifts in the IR spectra versus sodium alginate are evidence supporting that the desired metal-alginate complex has formed.

By observing the shift between asymmetric carboxyl (COO^-^) stretching frequencies, the change on metal co-ordination can be observed, thus indicating metal co-ordination:

**Table S4.**  ATR-FTIR peaks showing COO^-^ stretching

| Metal ion | Peak position / cm^-1^ | Peak position / cm^-1^ | Δυ peak / cm^-1^ |
| --- | --- | --- | --- |
| Na^+^ | 1593.3 | 1405.0 | 188.3 |
| Ba^2+^ | 1582.0 | 1409.2 | 172.8 |
| Ca^2+^ | 1596.2 | 1422.0 | 174.2 |
| In^3+^ | 1606.1 | 1400.7 | 208.2 |
| Zr^4+^ | 1604.7 | 1409.2 | 195.5 |

FTIR analysis indicated broad bands at 3370–3000 cm^−1^, representing bonded –OH groups. The band(s) observed at about 2700-2800 cm^−1^ are assigned to the –CH stretch. For indium and zirconium, a feature at 1725.1 and 1732.1 cm^-1^ respectively was noted, which was not present on the other samples. The cause of this is subject to an ongoing investigation.

The broad band at 1000-1035 cm^-1^ is assigned as the C-O stretching of both alcoholic and ether groups. It has been suggested that the shift of the alcoholic and ethereal groups is due to the weakening of the C-O bond in ethereal co-ordination in the case of Ca^2+^ with (in this case) a shift of 17 cm^-1^ ^[1]^. This effect is likely to increase with increasing ionic charge, however we observe an increased C-O bond stretching frequency suggesting either that the concentration of Zr and In in the samples are less than Ca^2+^ and Ba^2+^ or that ethereal and alcohol groups play a lesser role in binding tri- and tetravalent cations. A detailed concentration study is ongoing which seeks to examine these phenomena.

**Table S5.** ATR-FTIR peaks showing C-O stretching

| Metal ion | Peak position / cm^-1^ |
| --- | --- |
| Na^+^ | 1024.0 |
| Ba^2+^ | 1004.1 |
| Ca^2+^ | 1007.0 |
| In^3+^ | 1026.8 |
| Zr^4+^ | 1033.9 |


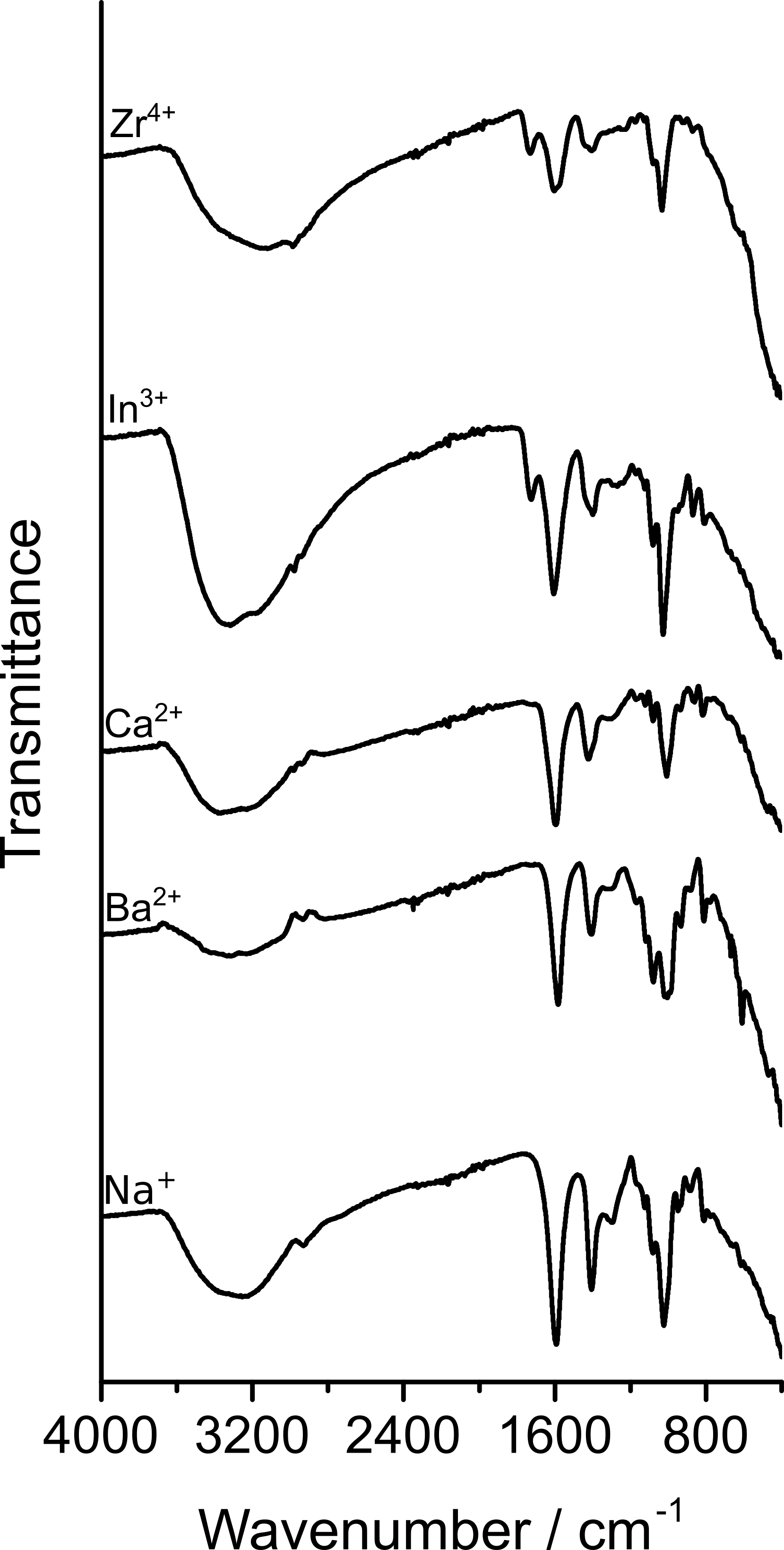


**Figure S3:** ATR-FTIR spectra of the metal alginate complexes.


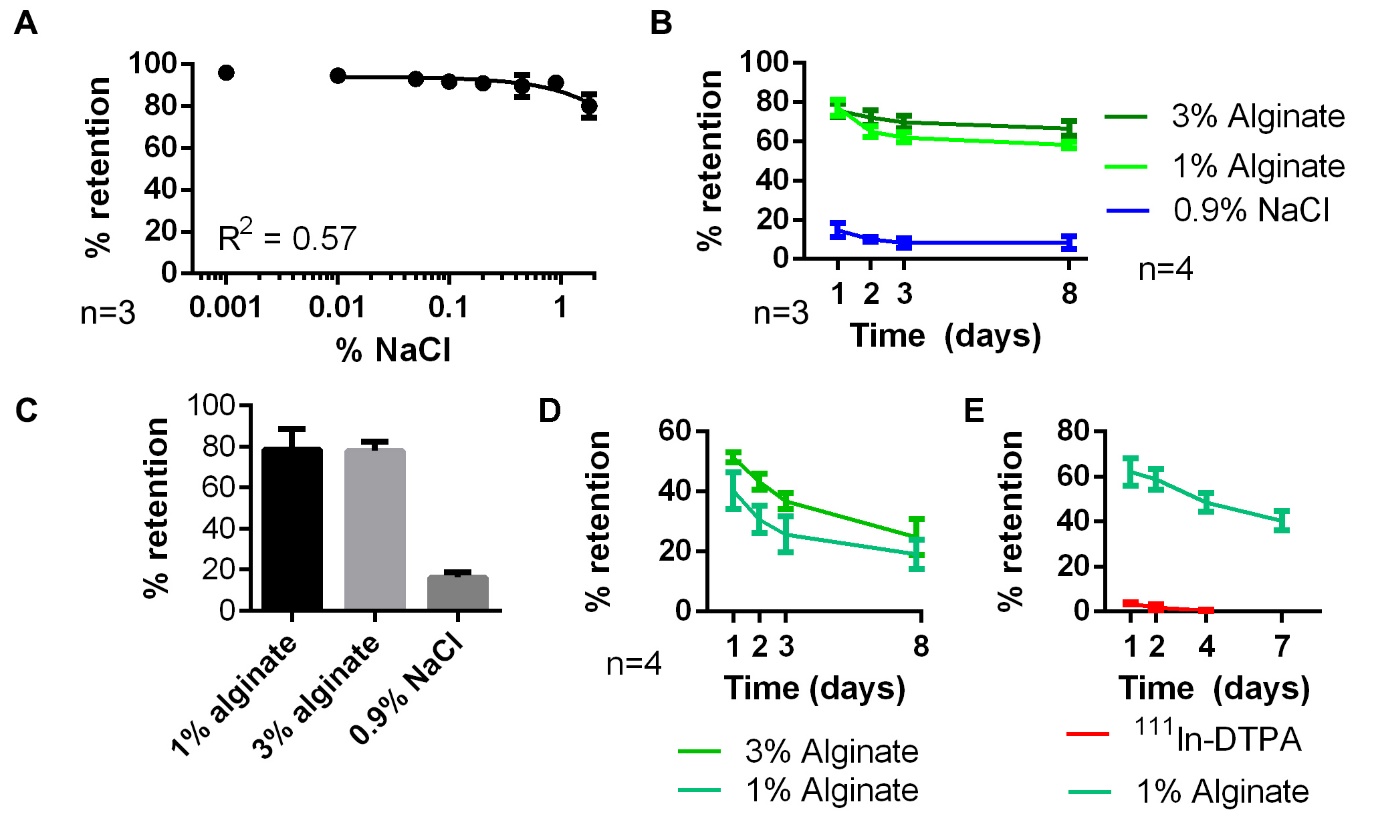
**Figure S4.** Retention of ^111^In after dialysis using a 8kDa molecular weight cut-off dialysis membrane. **A.** Retention of ^111^In in a 1% (w/v) solution of alginate (n=3) dialysed for 24 hours against NaCl solution at the indicated concentrations. This showed a weak trend towards lower retention with increasing salt concentrations. Line shows linear regression, R^2^=0.57, slope departs significantly from zero (p<0.0001). **B.** Retention of ^111^In in 1 and 3 % w/v alginate, and in a control 0.9% w/v (physiological) saline solution, all dialysed against a 0.9% saline solution for the indicated time (n=4). **C.** Dialysis of ^111^In alginate beads (cross-linked in 100mM CaCl_2_ for 4 minutes), for 24 hours against a 0.9% saline solution, showing greater retention compared to the negative control condition where the absence of alginate inside the dialysis membrane led to a much lower retention of ^111^In (16.18 % ± 2.52 SD; n=4), due to the lack of binding agent. **D.** Dialysis of ^111^In in 1 and 3% w/v alginate against foetal bovine serum (FBS), n=4. All points represent the mean, error bars show SD; some error bars are obscured by the data points. E. Dialysis of ^111^In in 1% w/v alginate and ^111^In-DTPA against human serum over 7 days.

**
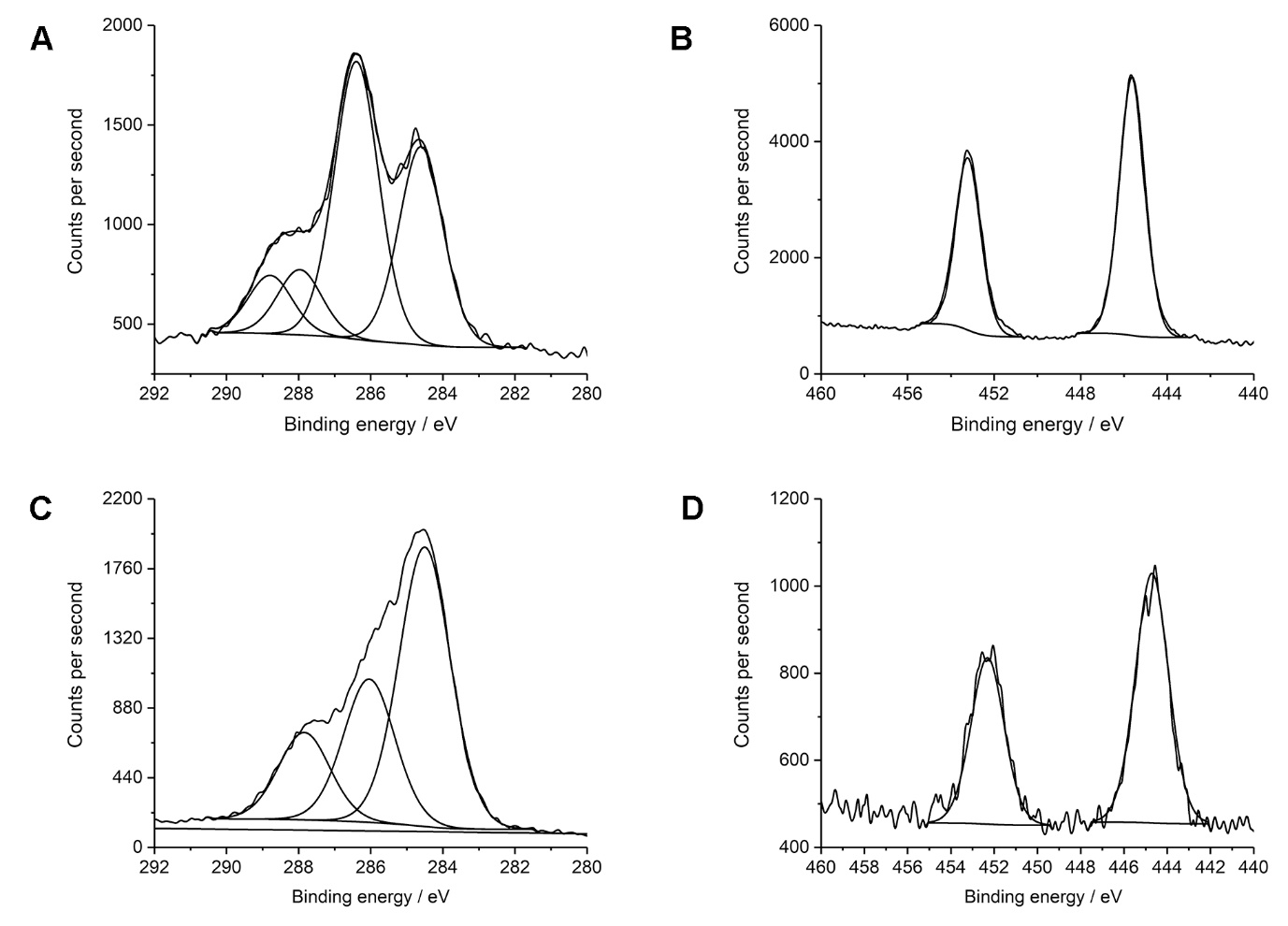
Figure S5. Fitted high resolution XPS spectra of C1s and In3d from In-crosslinked alginate beads at 4 and 10 days post implantation. A.** C1s and **B.** In3d spectra of In-alginate bead excised 4 days after subcutaneous implantation. Alginate carbon spectra closely match those of In-alginate pre-implantation (see figure S2D), while Indium is in its 3+ state. **C.** C1s and **D.** In3d spectra from In-alginate bead excised 10 days after subcutaneous implantation. Alginate carbon spectrum corresponds more closely with Ca-alginate than In-alginate (see figure S2C vs S2D), and Indium spectra were assigned to In_2_O_3_ ^[12]^.


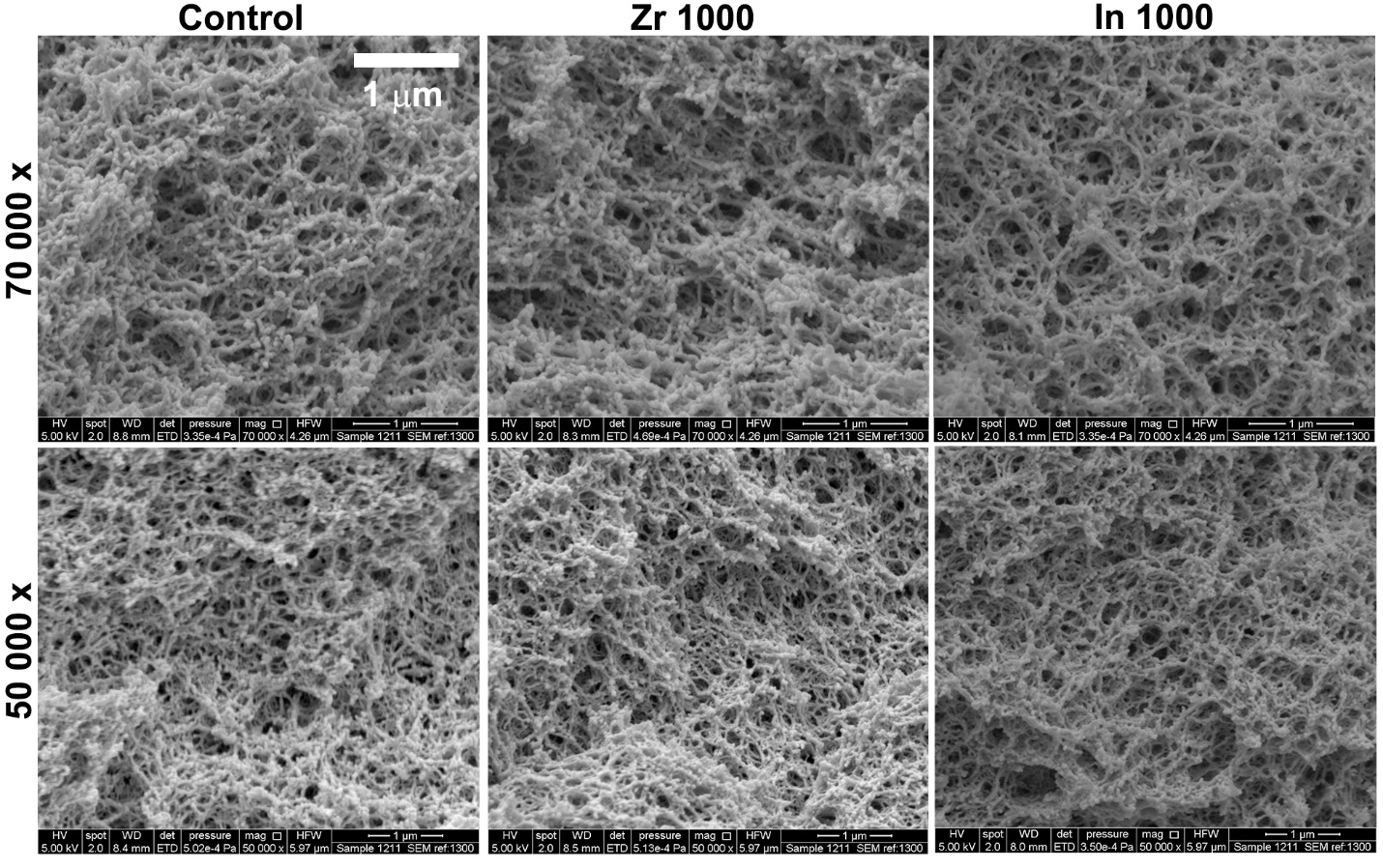


**Figure S6.** SEM images at 70000x and 50000x magnification, showing porosity of control hydrogels (3% alginate) cross-linked with 300mM calcium for 15 minutes, and otherwise identical preparations containing equivalent ZrCl_4_ and InCl_3_ doping to addition of 1000 MBq per mL of prepared alginate.


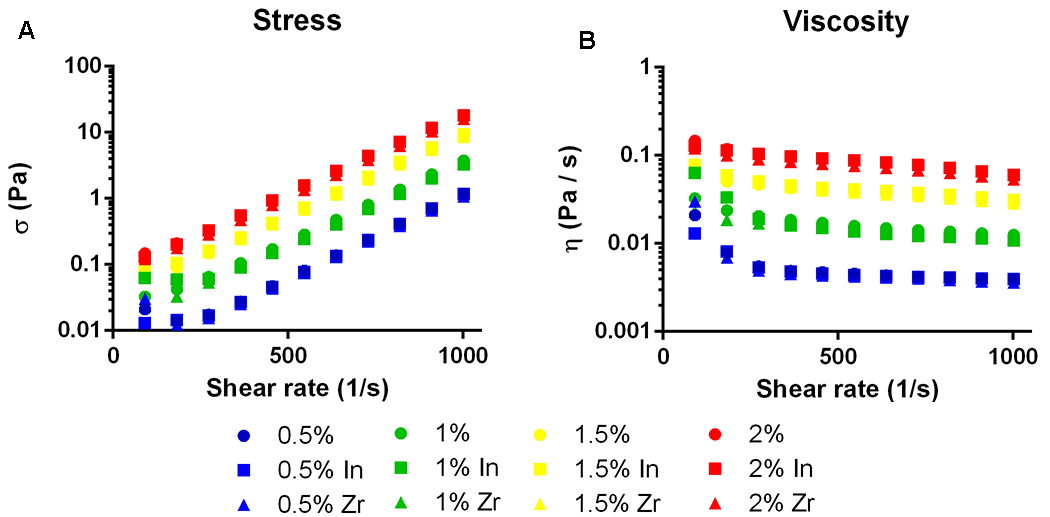


**Figure S7.** Effect on the Stress (σ) **(A)** and Viscosity (η) **(B)** of adding Indium (In) and Zirconium (Zr) at concentrations equivalent to 100MBq to varying concentrations (0.5% to 2%) of Sodium Alginate.


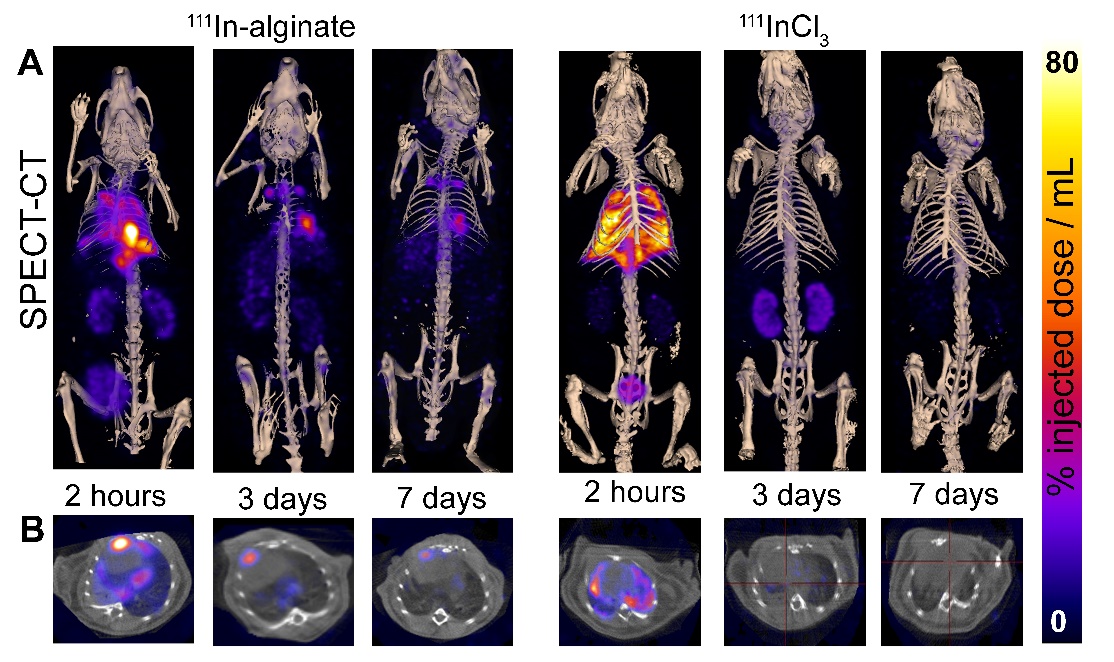


**Figure S8**. **A.** SPECT maximum intensity projections with 3D-rendered CT and **B.** axial SPECT-CT sections at 2 hours, 3 and 7 days post injection showing retention of ^111^In-alginate and rapid clearance of free ^111^InCl_3_ after ultrasound guided injection into the myocardium.

**
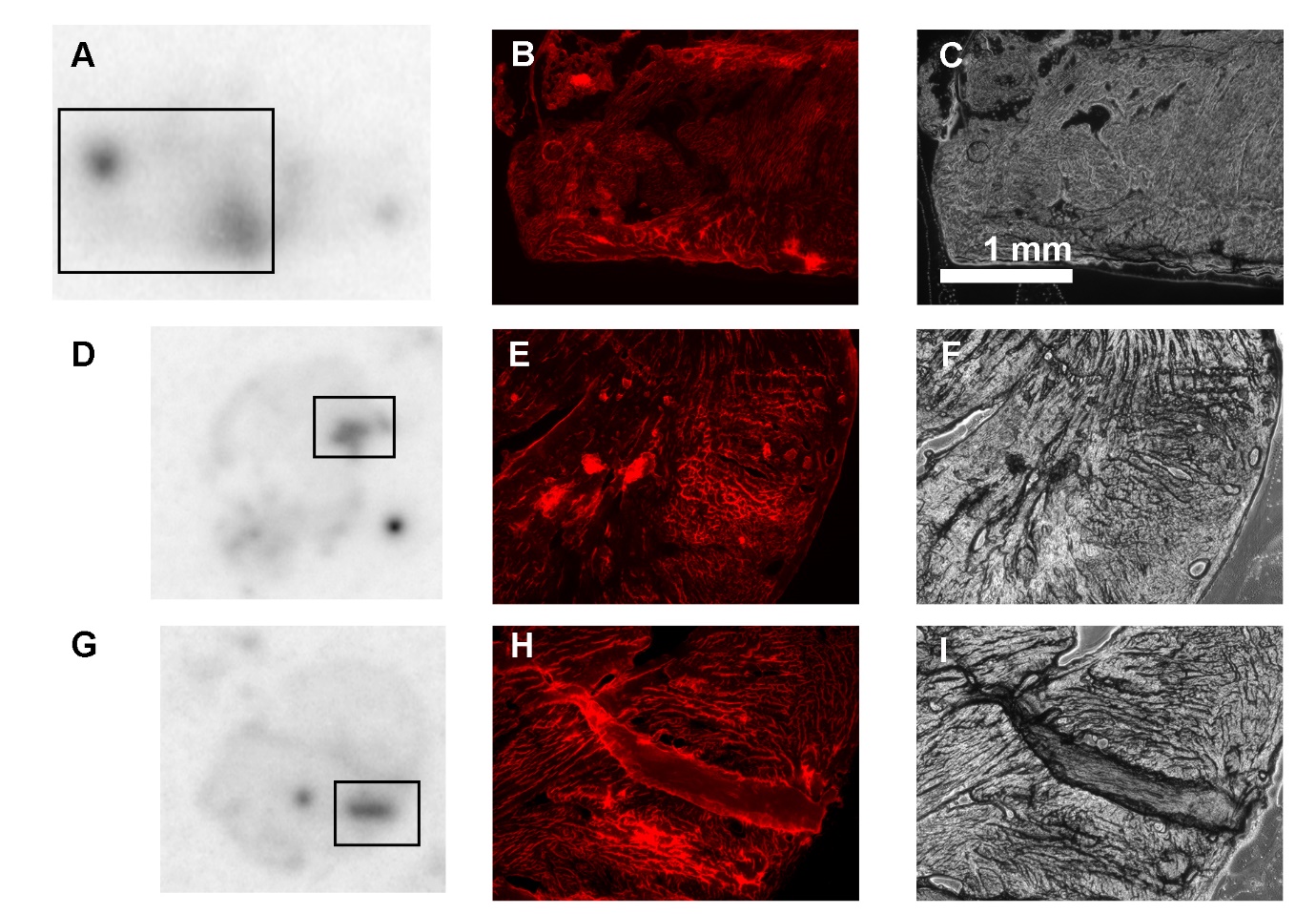
**

**Figure S9** Autoradiography (A,D,G) and matched fluorescence (B,E,H) and brightfield (C,F,I) images showing co-localisation of ^111^In and TRITC-alginate in the heart at 5 days post-implantation. Boxes on autoradiographs indicate the area magnified in the adjacent images.


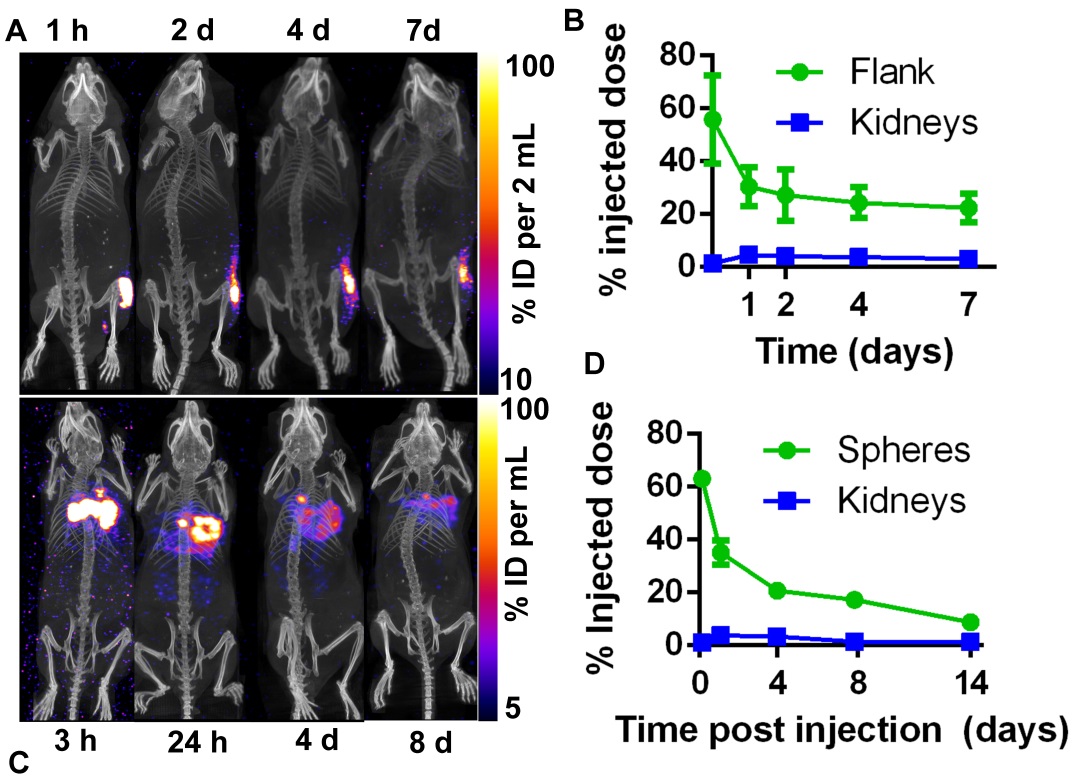


**Figure S10.** SPECT imaging of ^111^In-alginate shows retention of the hydrogel implanted in **(A)** the thigh muscle, and **(C)** cell-encapsulating beads subcutaneously implanted. Retention and degradation in **(B)** the thigh, and **(D)** the subcutaneous beads can be followed over the space of a week showing the location of the implanted material and clearance through the kidneys following breakdown. Points show the mean of n=3 animals, error bars show SD. At some points, error bars are obscured by the data points.


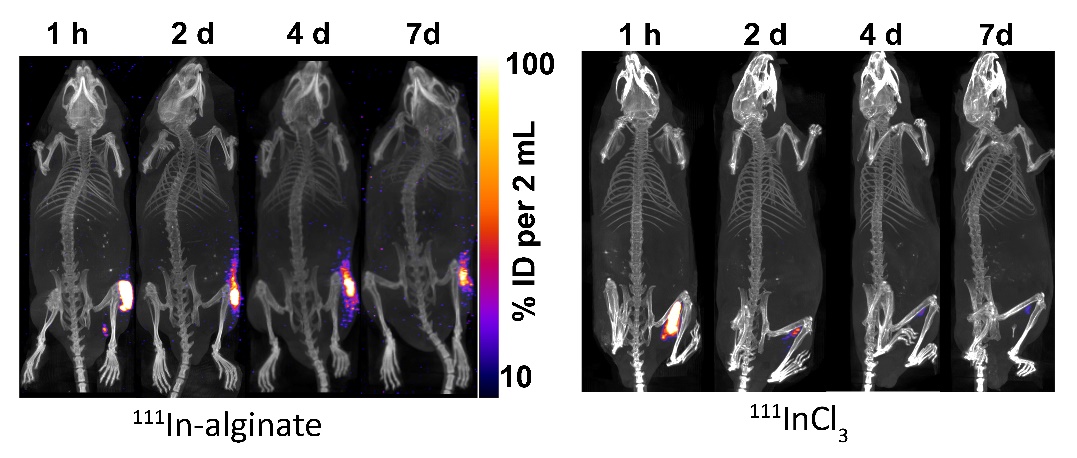


**Figure S11**. Maximum intensity projection SPECT-CT images showing the retention of ^111^In-algiante and ^111^InCl_3_ after ultrasound-guided injection into the hind flank muscle, from 1 to 7 days post injection.
**
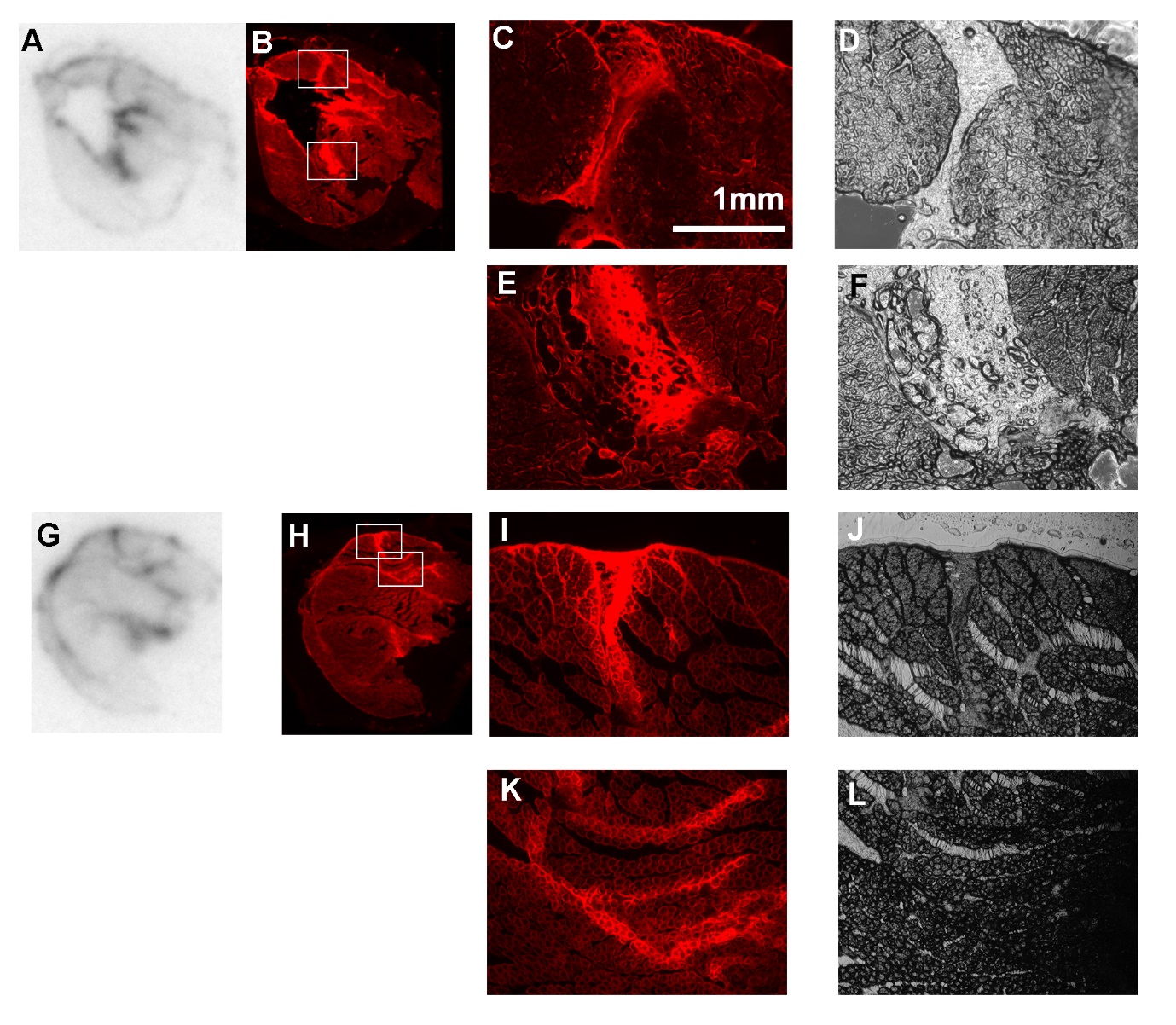
Figure S12. (A,G)** Autoradiography and **(B,C,E, H,I,K)** matched fluorescence and **(D,F,J,L)** brightfield microscopy images showing co-localisation of ^111^In and TRITC-alginate in the muscle at 5 days post-implantation. Boxes on the macroscopic fluorescence images (B,H) indicate the area magnified in the adjacent microscopy images.


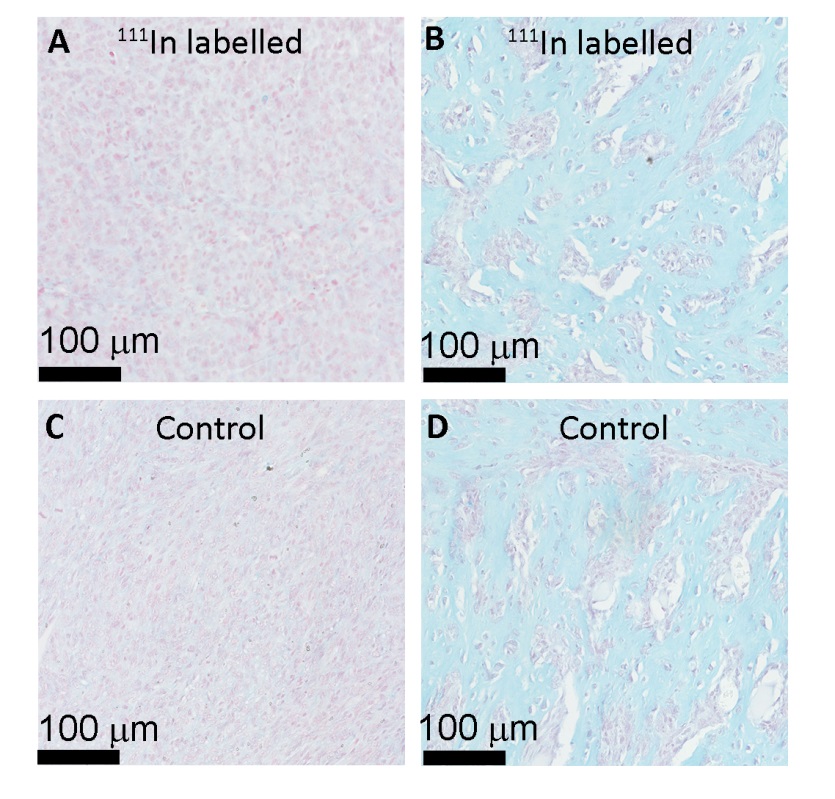


**Figure S13.** Histological sections stained with Alcian blue and nuclear fast red from **(A,B)** ^111^In-Alginate and **(C, D)** non-radiolabelled alginate spheres, showing (A,C) undifferentiated tissue and (B,D) differentiated cartilage from the cell mass formed the MSCs in the implanted alginate sphere.


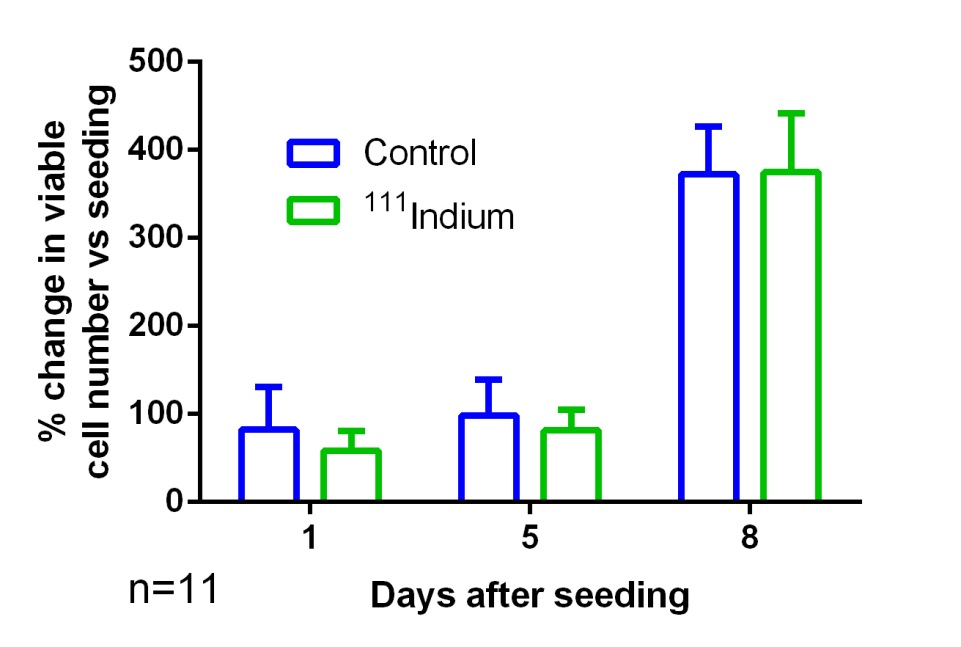


**Figure S14.** Mesenchymal stem cell growth in cross-linked (1% w/v) alginate beads with or without ^111^In. No significant difference was observed at any time point (2-tailed T-test). Columns represent the mean, error bars SD. Note that no growth was observed between days 1 and 5 while the alginate beads were intact. Upon observation at day 8, beads had partially disintegrated and cells had been able to proliferate across the bottom of the tissue culture plate well that contained the bead.

**
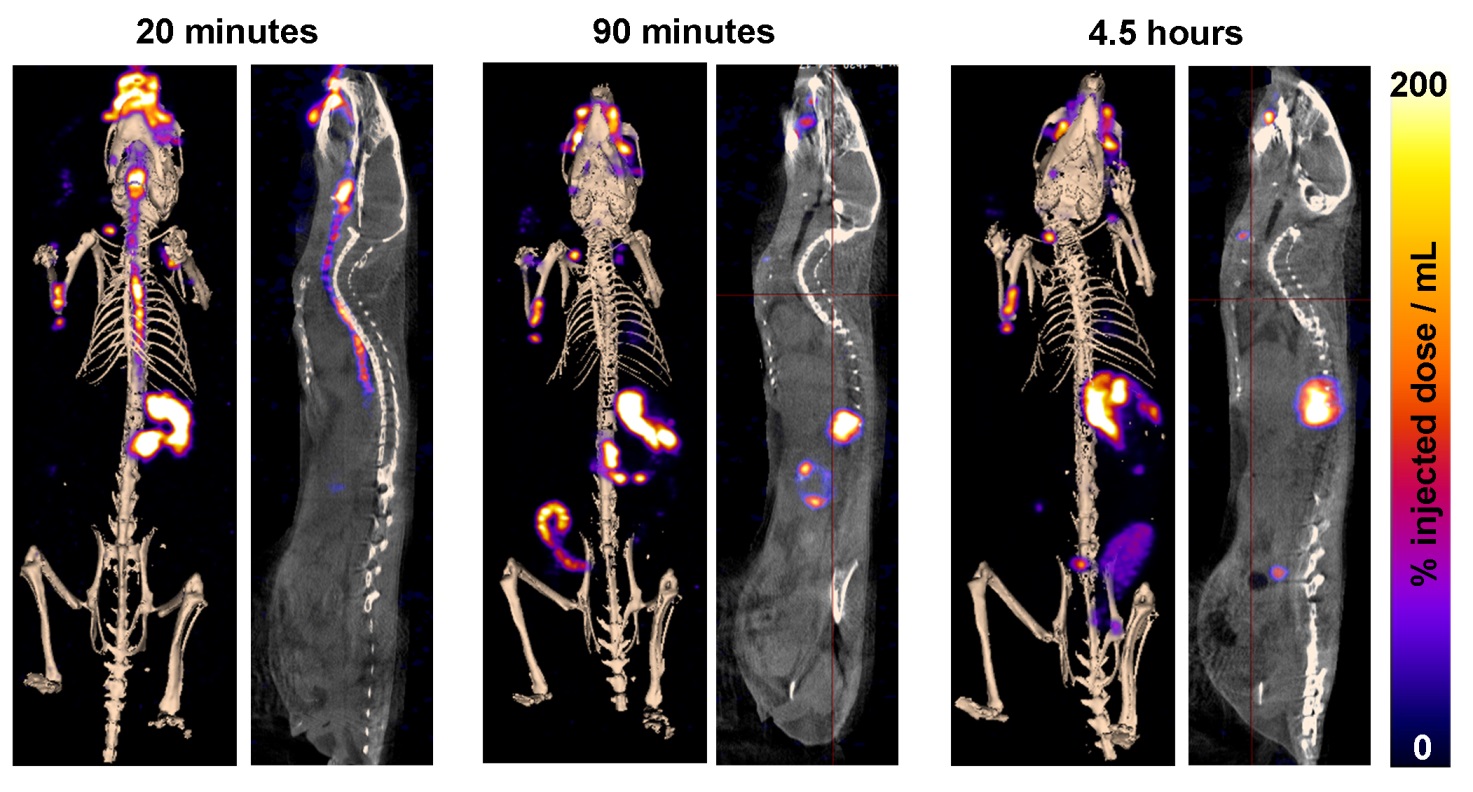
**

**Figure S15.** Maximum intensity projection SPECT-CT images and sagittal SPECT-CT sections showing ^111^In-Gaviscon biodistribution at the indicated time points after oral delivery. At the initial time point the material can be seen coating the oesophagus (~13%) and within the stomach, while at the later time points the amount in the oesophagus is negligible, and some material has passed into the intestines.

**
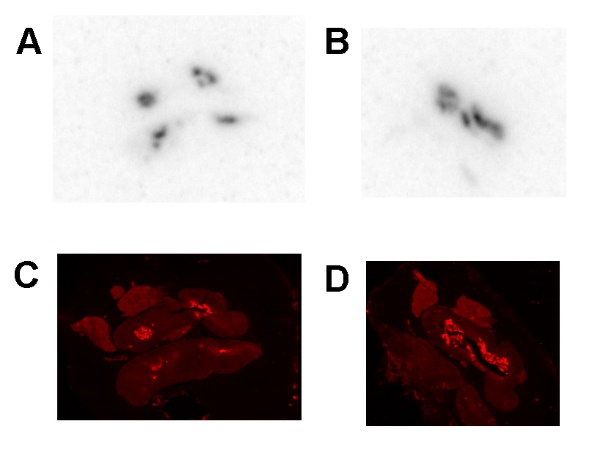
**

**Figure S16. (A,B)** Autoradiography and **(C,D)** matched fluorescence images showing co-localisation of ^111^In and gaviscon/TRITC-alginate in the small intestine at 75 minutes after oral dosing.


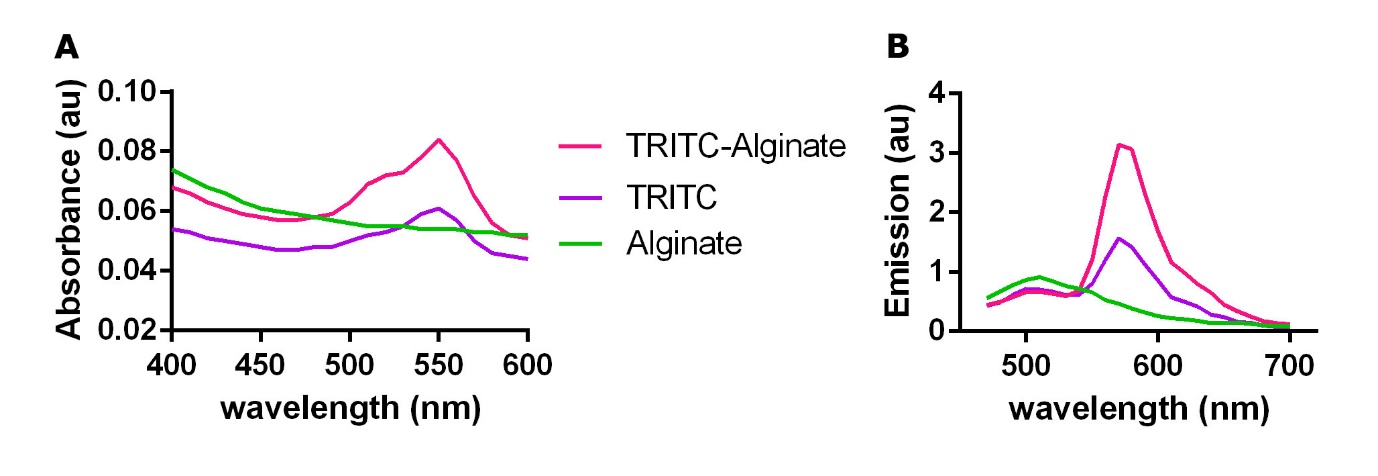


**Figure S 17. A.** Absorbance and **B.** Emission Spectra of TRITC-alginate, TRITC, and alginate, showing absorption maxima of ~550 nm and emission maxima of 570-580 nm for TRITC and TRITC-alginate, but not unlabelled alginate. Emission spectra were acquired using a 440 nm excitation wavelength.

Instrumentation and methods:

**Chemical Characterisation**

X-Ray photoelectron spectroscopy (XPS) was performed using a Thermo Scientific K-alpha spectrometer with monochromated Al Kα radiation, a dual beam charge compensation system and constant pass energy of 50 eV (spot size 400 μm). Survey scans were collected in the range 0–1200 eV. High-resolution peaks were used for the principal peaks of C (1s), Na (1s), Ba (3d), Ca (2p), In (3d) and Zr (3d). Peaks were modelled with CASA XPS software.

ATR-FTIR spectra were recorded on a Bruker ALPHA Platinum ATR single reflection diamond ATR between 400 cm^-1^ and 4000 cm^-1^.

**SEM sample preparation**

Alginate beads were prepared for SEM using a drop casting method with a 3% sodium alginate solution (w/v) and 300mM CaCl_2_ solution as cross-linker. Relevant molar equivalents of ZrCl_4_ or InCl_3_ were added to the alginate solution prior to crosslinking with calcium, assuming specific activities of 0.15 GBq/nmol and 1.85 GBq/nmol respectively. Following 15 minutes of crosslinking, samples were changed through an ethanol series (50%, 70%, 90%, 100%; 30 minutes per incubation), then stored in a fresh solution of 100% ethanol before drying in a Quorum K850 critical point dryer. Samples were then fractured under liquid nitrogen, and given a 5 nm gold coating in a Quorum Q150T Sputter coater. Images were obtained at 50 000x and 70 000x magnification using a FEI Quanta 200 FEG ESEM (ThermoFisher Scientific).

**Synthesis of beads, microbeads, and sheets**

Hydrogel microbeads were formed by aerosolising a solution of 0.5 to 3% alginate solution (w/v) containing the desired amount of ^111^InCl_3_ or ^89^ZrCl_4_ using a spray bottle (Travel spray bottle, Boots) directed into a cross-linking solution of 300 mM CaCl_2_. Cross-linking was allowed to proceed for 3 minutes before straining meshes (100 μm; Corning Falcon™ Cell Strainers) were used to filter out beads above 100 μm in diameter.

Hydrogel beads were formed by drop-casting 0.5 to 3% alginate solution (w/v) containing the desired amount of ^111^InCl_3_ or ^89^ZrCl_4_ into a cross-linking solution of 100 to 300 mM CaCl_2_ or BaCl_2_. Cross-linking was allowed to proceed for 3 to 10 minutes. Bead size was controlled by altering the volume of alginate dispensed into the cross-linking bath.

Hydrogel sheets were synthesised on filter paper (Whatman filter paper No. 1) cut into templates of the chosen size and shape. These were pre-soaked for 10 minutes in CaCl_2,_ before alginate solutions (0.5% to 3% w/v) mixed with the desired amount of ^111^InCl_3_ or ^89^ZrCl_4_ were spread over the top of the filter paper at the desired thickness and left for 10 minutes to cross-link, before the template was removed.

**Viscosity measurements**

Viscosity measurements were made on 1 mL samples over a shear rate range of 90 to 1000 s^−1^ using a Bohlin CVO rheometer (Malvern Instruments). This was set up in the cone and plate geometry (60 mm, 0.995º), and the temperature maintained at 20 ± 0.05 degrees. A gap of 25 μm was used for all experiments.

**Radioactivity Measurements**

All radioactivity measurements were made using either a dose calibrator (Capintec Curimentor), or where appropriate an automated high-sensitivity gamma counter (Perkin Elmer Wizard^2^). Both instruments were calibrated for use with the respective isotopes and appropriately shielded from background radiation. For the Perkin Elmer Wizard, to convert counts into MBq, a dose calibration curve was set up using a serial dilution of known activity amounts. For *in vivo* analysis of activity, regions of interest were drawn manually around sites of injection using the CT data for anatomical reference, using VivoQuant software (Invicro).

**Cell culture and growth Assay**

For the in vitro growth assay luciferase-expressing D1 ORL UVA (ATCC® CRL-12424™) mouse mesenchymal stem cells (a kind gift from Dr Arthur Taylor, University of Liverpool) were suspended in a 1.5% (w/v) alginate solution at a concentration of 20 million per mL. For the Indium condition, ^111^InCl_3_ was added to the alginate solution at 30 MBq per mL final concentration. Crosslinking was done for 10 minutes using a 100 mM CaCl_2_ solution, with a bead volume of 100 μL. Beads were cultured in individual wells of an opaque multi-well cell culture dish in grown in DMEM (Invitrogen, UK), supplemented with 10 % fetal bovine serum (FBS), 2 mM *L*-glutamine and 0.1 mM MEM nonessential aminoacids. This was incubated at 37 ºC with 5 % CO_2_ and 95 % humidity. Viability was measured 6 minutes after the addition of luciferin to the growth media (final concentration of 50 μg/mL), using a platereader (Varioskan Lux, Thermo-Fisher), with acquisition time of 1000 ms. Growth media was changed following each assay reading. A total of 11 independently synthesised beads were used for each condition.

**Autoradiography**

For co-registration with fluorescence images, excised hearts, muscles, and intestines were frozen in Optimal Cutting Temperature solution (OCT; Cellpath), and sectioned at 20 µm using a cryotome (Leica CM3050S) onto slides (SuperFrost; ThermoScientific). Fluorescence microscopy was done using an EVOS XL (Thermofisher) equipped with RFP filters (Ex. 531nm, Em. 593nm), and whole slide fluorescence images were obtained at 50 μm resolution (Ex. 532nm, Em. 580) using a Typhoon 9410 Imager (GE Healthcare). The same slides were then exposed to a storage phosphor screen (GE Healthcare) for between 10 minutes and 3 days to obtain sufficient signal, which was scanned at 50 μm resolution (Typhoon 9410 Trio + Imager), and the resulting images were processed by ImageJ (NIH, USA).

For co-registration with bioluminescence images, mice were sacrificed, the chest was opened and the hearts flushed with high potassium (30mmol) heparinised saline *via* the left ventricle. Hearts were then excised, flash frozen, then cut into 1mm thin sections along the short axis, before incubation with 1% TTC for 30 minutes. Sections were mounted on microscope slides, photographed and exposed to unmounted GP 20 × 25 cm^2^ phosphor screens (VWR international LTD, U.K.) for 2 hours. After this time, the screens were scanned (Typhoon 9410 Trio+, GE Healthcare, U.K.) at 25 μm resolution with an acquisition time of ~ 2 h 30 min, and the resulting images were processed by ImageJ (NIH, USA).

**Alginate Dye-labelling**

Alginate was labelled with tetramethylrhodamine (TRITC; Excitation maximum = 543 nm; Emission maximum = 569 nm) using an EDC/NHSS conjugation protocol adapted from Zhu et al ^[13]^. Sodium alginate (500 mg) was dissolved in 20 mL sodium acetate buffer (pH 5, 100 mM), and mixed with 210 mg N-(3-Dimethylaminopropyl)-N′-ethylcarbodiimide hydrochloride (EDC) and 125 mg N-Hydroxysulfosuccinimide sodium salt (NHSS) and reacted for 30 minutes at room temperature, before addition of 250 mg diaminohexane and 4 hours further incubation. The mixture was precipitated by addition of 10 mL 2-propanol, and resuspended in 20 mL sodium bicarbonate buffer (pH 8.5) with 2 mg TRITC (mixed isomers, Sigma Aldrich) and incubated for 4 hours at room temperature, before precipitation with 10 mL acetone. The precipitate was then washed 3 times overnight in methanol to remove unreacted TRITC, and resuspended in 0.9 % w/v NaCl solution. To confirm labelling, the fluorescence spectrum of the labelled alginate was then measured using a microplate reader (VarioSkan Lux; ThermoFisher) with comparison to a TRITC dye solution and unlabelled alginate (see figure S15).

**Histology**

Alginate spheres were excised at 20 days post implantations, at which point teratoma formation had occurred. Tissues were fixed overnight in 4% paraformaldehyde then changed into 70% ethanol. Samples were dehydrated and paraffin embedded before sectioning at 20 μm thickness onto glass slides. For Alcian blue staining, slides were deparaffinised and rehydrated through an ethanol series before staining in Alcian blue solution (1% Alcian Blue solution in 3% Acetic acid, pH 2.5, Sigma Aldrich product: B8438-250ML) for 15 minutes. Slides were then rinsed under running tap water for 2 minutes before counterstaining in nuclear fast red solution (Sigma Aldrich product: N3020-100ML) for 5 minutes. Samples were then rinsed again under running tap water for 1 minute before dehydrating in an increasing ethanol series and finally in 100% Xylene, before coverslipping.

**Preparation of ^89^ZrCl_4_**

^89^ZrCl_4_ was obtained from ^89^Zr-oxalate stock provided by the supplier (Perkin Elmer) following an established method ^[14]^. A strong anion exchange cartridge (Waters, Sep-Pak Acell Plus QMA Plus Light Cartridge, 130 mg sorbent) was pre-washed with 6mL methanol followed by 10 mL 0.9% saline and 10 mL HPLC-grade water. This was then loaded with between 20 and 100 μL (5 to 50 MBq) ^89^Zr in 1M oxalate solution (Perkin Elmer) diluted with HPLC-grade water to 500 μL. This was then washed with a further 40 mL of HPLC-grade water and eluted with 100 to 400 μL of HCl (1M). Typically 80 to 85 % of the initial activity was obtained in the HCl elution with the majority of the remainder left on the column and not retrievable by subsequent elution.

**Activity retention**

Alginate (1% sodium alginate (w/v) in distilled water) was added to 100 μL aliquots containing 0.2 to 0.5 MBq of either ^111^InCl_3_ or ^89^ZrCl_4_ in HCl (1M) to a final volume of 1mL, and loaded into dialysis membranes (BioDesignDialysis Tubing™ D112, 15.5mm diameter, 8 kDa molecular weight cut off; Thermofisher Scientific). Samples were dialysed against 40 mL of either distilled water, 2.5mM DTPA (pH 7.4), Foetal Bovine Serum (FBS; Gibco), Human Serum (Standard Pooled Human Serum, Cambridge Bioscience), or 0.001 to 1.8 % NaCl. After 24 hours, and at timepoints thereafter, membranes and dialysis media were separated and counted for radioactivity, and the percentage of activity retained in the membrane was calculated.

***In vivo* animal work**

All animal studies were approved by the University College London Biological Services Ethical Review Committee and licensed under the UK Home Office regulations and the Guidance for the Operation of Animals (Scientific Procedures) Act 1986 (Home Office, London, United Kingdom). All animal methods were performed in accordance to institutional ethical guidelines and regulations.

**Heart:** For intramyocardial delivery, 10 week old, male C57Bl/6 mice (n = 3) were anaesthetised with 2% isoflurane in 100% O_2_. Hearts were visualised using ultrasound (Visualsonics Vevo 2100) and 50 µl of 2% ^111^In alginate with or without MSCs (4x10^7^ cells/mL alginate) was injected into the anterolateral wall of the myocardium using ultrasound guidance of a 30 gauge needle. Ten days later, hearts were flushed with saline, excised and sliced for autoradiography and *ex vivo* BLI. For *ex vivo* studies of fluorescence / autoradiography, the above procedure was repeated using TRITC-alginate in place of alginate.

**Muscle:** For muscle delivery, 10 week old, male C57Bl/6 mice (n = 3) were anaesthetised with 2% isoflurane in 100% O_2,_ and 50 µl of 2 % ^111^In-alginate was injected into the left thigh muscle using a 30 gauge needle. For *ex vivo* studies of fluorescence / autoradiography, the above procedure was repeated using TRITC-alginate in place of alginate.

**Subcutaneous beads:** For subcutaneous bead implantation prior to *in vivo* imaging, 2 mm diameter, ^111^In alginate beads were fabricated by dropcasting of 2% (w/v) alginate pre-mixed with ^111^InCl_3_ to a final concentration of 20 million mouse mesenchymal stem cells/mL into a bath of 100mM CaCl_2_ and left to cross link for 4 minutes. 10 week old, male C57Bk6 mice (n = 3) were anaesthetised with 2% isoflurane in 100% O_2,_ Fur was removed from the dorsal region, the skin was sterilised and a small incision was made between the shoulder blades. Beads (6 to 7 beads per animal) were implanted into the subcutaneous space and the incision was closed with a suture.

For implantation prior to XPS analysis, 3% w/v alginate was drop cast into a bath of 300 mM InCl3 and left to cross link for 10 minutes, washed three times in 0.9 % w/v saline, and implanted as above.

**Oral:** For oral administration of ^111^In-labelled anti-acid treatment, Gaviscon™ Original Aniseed (Boots Plc, Tottenham Court Road, England) was mixed with ^111^InCl_3_ at a ratio of 5MBq per 100 μL of Gaviscon. Original Aniseed flavour was selected as rodents have a strong preference for the smell of aniseed, and will preferentially consume anise-flavoured food over control food ^[15]^, whereas they have a strong aversion to peppermint scent ^[16]^. 10 week old, male C57Bk6 mice (n = 2) were scruffed and voluntarily consumed the ^111^In-Gaviscon (100 μL) which was dispensed from a pipette tip positioned next to the mouth. Mice were then imaged with SPECT-CT at 20 minutes, 90 minutes, and 4.5 hours (270 minutes) after administration. For *ex vivo* autoradiography/fluorescence experiments, mice were fasted overnight to reduce the dietary source of autofluorescence from the gastro-intestinal tract before administering a 3:1 mixture of Gaviscon and TRITC-alginate (3% w/v) as above.

**Nasal:** For nasal dosing of ^111^In-alginate, a solution was prepared to a final concentration of 0.6% w/v alginate containing 150 MBq/mL ^111^InCl_3_. Of this, 20μL was administered to each of the left and right nasal passages using a micropipette (Gilson) while the animal was scruffed, resulting in doses of ~6 MBq ^111^In per animal. 10 week old, male C57Bk6 mice (n = 3) were used per condition. As a control, mice were administed an equal volume of ^111^In-EDTA (Ethylenediaminetetraacetic acid; Sigma Aldrich) was made prepared by mixing a 1mM EDTA solution (pH 7.4) with ^111^InCl_3_ to a final activity of 150 MBq/mL. Thin layer chromatography using a 0.9% saline mobile phase was used to confirm binding of ^111^In to EDTA before administration; unbound ^111^In will remain at the origin while ^111^In-EDTA will migrate with the solvent front. For nasal dosing of 0.6% alginate containing ^123^I-DatScan (20 MBq/100μL), 20 μL was dosed per nostril using a micropipette and SPECT-CT imaging was performed at 2 hours post-dosing.

For MRI nasal dosing of Mn-alginate was done using a micropipette to administer 20 μL of a 0.6% solution of alginate (w/v) containing 10mM MnCl_2_. Imaging was performed at 30 minutes post dosing.

**In vivo imaging**

During all *in vivo* imaging, mice were maintained at 37 ºC under isofluorane breathable anaesthesia (1 to 2%) in oxygen. A small animal physiological monitoring system (SA Instruments, Stony Brook, NY) was used to monitor core body temperature and respiration rate.

SPECT data was acquired using a NanoScan SPECT-CT (Mediso) using 1.4mm pinholes, interfaced to a computer running Interview Fusion software (Bartec). CT images were acquired using a 55 kVP X-ray source, 500 ms exposure time, 180 projections, a pitch of 1.5, and a total scan time of 3 minutes 45s. SPECT Images were obtained using a 4-head scanner with nine 1.4 mm pinhole apertures in helical scan mode using a time per projection of 60s resulting in a scan time of 40 minutes. CT images were reconstructed in voxel size 124 x 124 x 124 µm, whereas SPECT images were reconstructed in a 256 × 256 matrix prior to being overlayed. Images were reconstructed using HiSPECT software, and analysed using VivoQuant software (Invicro).3D ROIs were manually drawn around the liver, lungs, and kidneys, and used to calculate the percentage of injected dose/organ (%ID/organ).

PET data was acquired using a NanoScan PET-CT (Mediso) interfaced to a computer running Interview Fusion software (Bartec), which was also used to reconstruct images. Data was analysed using VivoQuant software (Invicro).

MRI data was acquired using a 9.4T horizontal bore scanner (Varian) interfaced to a VNMRJ 3.1 (Varian) imaging console, using an Agilent 205/120HD gradient set, 72 mm inner diameter volume coil for RF transmission (Rapid Biomedical), and a 2 channel array surface coil (Rapid Biomedical) for signal reception. T_1_-weighted images were acquired using a multi slice gradient echo (TE 2.5 ms/ TR 75 ms; 75 degree flip angle), with a 100x100x250 μm voxel size.

*In vivo* Bioluminescence imaging was performed using an IVIS 200 (Perkin Elmer) imaging device at 10 minute post intraperitoneal injection of D-luciferin (Promega) solution at 150 mg/kg per mouse. *Ex vivo* imaging was performed immediately after cervical dislocation and dissection of the animal.

**References**

[1] A. Jejurikar, X. T. Seow, G. Lawrie, D. Martin, A. Jayakrishnan, L. Grondahl, *Journal of materials chemistry* **2012**, *22*, 9751-9758.

[2] J. Szepvolgyi, A. Tudos, I. Bertoti, *J Electron Spectrosc* **1990**, *50*, 239-250.

[3] H. Vandoveren, J. A. T. Verhoeven, *J Electron Spectrosc* **1980**, *21*, 265-273.

[4] R. P. Vasquez, *J Electron Spectrosc* **1991**, *56*, 217-240.

[5] B. Demri, D. Muster, *J Mater Process Tech* **1995**, *55*, 311-314.

[6] M. I. Sosulnikov, Y. A. Teterin, *Dokl Akad Nauk Sssr+* **1991**, *317*, 418-421.

[7] M. Pessa, A. Vuoristo, M. Vulli, S. Aksela, J. Väyrynen, T. Rantala, H. Aksela, *Phys Rev B* **1979**, *20*, 3115-3123.

[8] C. D. Wagner, *Handbook of X-Ray and Ultraviolet Photoelectron Spectroscopy. D. Briggs, Editor* **1977**.

[9] D. Majumdar, D. Chatterjee, *Journal of applied physics* **1991**, *70*, 988-992.

[10] C. Sleigh, A. P. Pijpers, A. Jaspers, B. Coussens, R. J. Meier, *J Electron Spectrosc* **1996**, *77*, 41-57.

[11] C. Sartori, D. S. Finch, B. Ralph, *Polymer* **1997**, *38*, 43-51.

[12] D. T. Clark, T. Fok, G. G. Roberts, R. W. Sykes, *Thin Solid Films* **1980**, *70*, 261-283.

[13] H. Zhu, R. Srivastava, J. Q. Brown, M. J. McShane, *Bioconjugate chemistry* **2005**, *16*, 1451-1458.

[14] J. P. Holland, Y. Sheh, J. S. Lewis, *Nuclear medicine and biology* **2009**, *36*, 729-739.

[15] G. Jokic, M. Vuksa, S. Djedovic, B. Stojnic, D. Kataranovski, T. Scepovic, *Pesticidi i fitomedicina* **2013**, *28*, 111-116.

[16] R. E. Brown, J. A. Willner, *Behav Neural Biol* **1983**, *38*, 251-260.
